# Childhood and adolescent nutrition outcomes among girls exposed to gender-based violence: A rapid evidence assessment of quantitative research

**DOI:** 10.1371/journal.pone.0281961

**Published:** 2023-02-16

**Authors:** Luissa Vahedi, Manuela Orjuela-Grimm, Silvia Bhatt-Carreno, Sarah Rachel Meyer

**Affiliations:** 1 Brown School, Washington University in St. Louis, St. Louis, Missouri, United States of America; 2 Department of Epidemiology and Pediatrics, Columbia University Irving Medical Center, New York City, New York, United States of America; 3 Department of Epidemiology, Columbia University, New York City, New York, United States of America; 4 Institute for Medical Information Processing, Biometry, and Epidemiology, Ludwig-Maximilians-Universität, Munich, Germany; Tabba Heart Institute, PAKISTAN

## Abstract

**Background:**

An emerging evidence base has explored the nutritional consequences of gender-based violence (GBV) perpetrated against girls during childhood/adolescence. We conducted a rapid evidence assessment of quantitative studies describing associations between GBV and girls’ nutrition.

**Methods:**

We adapted systematic review methods and included empirical, peer-reviewed studies, published after 2000 (until November, 2022), that were written in Spanish or English and reported quantitative associations between girls’ exposure to GBV and nutrition outcomes. A variety of GBV forms were considered: childhood sexual abuse (CSA), child marriage, preferential feeding of boys, sexual IPV and dating violence. Nutrition outcomes included anemia, underweight, overweight, stunting, micronutrient deficiencies, meal frequency, and dietary diversity.

**Results:**

In total, 18 studies were included, 13 of which were conducted in high-income countries. Most sources utilized longitudinal or cross-sectional data to quantify associations between CSA, sexual assault, and intimate partner/dating violence and elevated BMI/overweight/obesity/adiposity. Findings suggest that CSA perpetrated by parents/caregivers is associated with elevated BMI/overweight/obesity/adiposity via cortisol reactivity and depression; this relationship may be compounded by additional intimate partner/dating violence in adolescence. The effects of sexual violence on BMI are likely to emerge during a sensitive period of development between late adolescence and young adulthood. Emerging evidence was found regarding the relationship between child marriage (and the related exposure: age at first pregnancy) and undernutrition. The association between sexual abuse and reduced height and leg length was inconclusive.

**Conclusion:**

Given that only 18 studies were included, the relationship between girls’ direct exposure to GBV and malnutrition has received little empirical attention, especially with respect to studies conducted in LMIC and fragile settings. Most studies focused on CSA and overweight/obesity, where significant associations were found. Future research should test the moderation and mediation effects of intermediary variables (depression, PTSD, cortisol reactivity, impulsivity, emotional eating) and consider sensitive periods of development. Research should also explore the nutritional consequences of child marriage.

## Introduction

Childhood (defined as the time between infancy to the onset of puberty) and adolescence (between childhood and culturally/socially defined adulthood) are sensitive periods of growth and development that lay the foundation for later health status and health behaviors [1–3]. Adverse childhood experiences (ACEs) are traumatic events that occur during childhood and adolescence and undermine a child’s physical, social, and emotional development, learning, and the sense of safety and stability [4]. ACEs can have a profound effect on later development. Examples of ACEs include witnessing or experiencing abuse or violence (such as childhood sexual abuse [CSA]), the incarceration of a parent/guardian, and having parents/guardians with substance use or mental health problems [5]. Exposure to ACEs can contribute to harmful levels of stress hormones that can negatively affect brain development and immune responses [6–8]. In particular, ACEs have been studied as primary exposures contributing to adult morbidity and mortality [4]. A rich body of longitudinal research has investigated relationship between ACEs and adult health. ACEs are directly associated with a greater likelihood of chronic diseases (type 2 diabetes and cardiovascular disease), mental health outcomes (depression and attempted suicide), sexually transmitted infections, and substance abuse ranging from nicotine to intravenous drugs [9–17].

Interpersonal violence is the intentional use of physical force or power against another person to enact physical, sexual, psychological harm, including neglect or deprivation [18]. Recent estimates indicate that interpersonal violence is the fifth leading cause of death among youth 10–24 years old, representing 3.5% of disability adjusted life years globally [19]. However, the distribution of interpersonal violence and its forms is not random. In particular, girls are disproportionately affected by gender-based violence (GBV) [20], such as dating violence/intimate partner violence (IPV) (inclusive of physical, emotional, sexual, economic and controlling behaviors); GBV is defined as the intentional or threatened use of power or physical force, against another person or group of people based on their perceived gender or gender identity [20].

In addition to dating violence/IPV, GBV also includes CSA, child marriage, female genital cutting, and marital rape [21]. GBV can be perpetrated by intimate partners, family members, peers, and authority figures, and is underscored by a gender power hierarchy, wherein “masculinity” is privileged over “femininity” and cis-gender identities are presumed to be natural and superior in comparison to trans-identities [22]. Globally, 10% of females under the age of 20 have been forced to perform sexual acts [23], while 25% of young women who have been in relationships experience IPV by their mid-twenties [24, 25]. In sub-Saharan Africa, the prevalence of child marriage (defined as informal unions/marriages between a child under the age of 18 and another child or adult) is estimated to be 54.0%, ranging from 16.5% to 81.7% [26].

GBV perpetrated against females during childhood and adolescence is a specific ACE subtype (although not the only form of ACE) that can affect growth, development, and health [27]. Studies of ACEs have improved the knowledge base concerning the health impacts of GBV exposure during childhood. However, ACE studies don’t disentangle GBV from other ACEs and sometimes lack sex disaggregation. Further, the majority of research exploring the health sequala of GBV focuses on violence against women specifically and examines adult morbidity specifically [28–30] rather than focusing on exposures and outcomes that can be measured during childhood and adolescence. Further, even when CSA is the primary interest variable, health outcomes are often measured in adulthood. Overall, there is less research exploring the childhood and adolescent health consequences of GBV perpetrated against females during childhood and adolescence.

Nutrition, a critical contributor to health and wellbeing, is defined as the process of taking in food for growth, metabolism, and repair [31]. Moreover, childhood nutritional status can impact health outcomes across the life course, with micro nutrient insufficiencies such as iron or calcium contributing to cognitive impairments and osteopenia later in life [32]. Research examining the link between GBV exposure and nutrition has predominantly focused on forms of GBV perpetrated against adult women during and immediately after pregnancy [33–35]. For example, IPV victimization during pregnancy and lactation negatively affects breastfeeding practices and has been linked to premature birth and labor and low birth weight, known risk factors for later childhood low height and weight for age [36]. The evidence base exploring the impact of GBV on childhood nutrition outcomes has focused on GBV perpetrated against mothers and nutrition outcomes among their children [36–38] (Authors, under review). Less understood are the childhood and adolescent nutritional consequences of GBV among girls who have themselves experienced GBV during childhood and adolescence.

Emerging research indicates that some nutrition-related disorders of adulthood may begin to manifest during childhood and adolescence and that exposure to GBV may contribute to the development of these disorders. Research has postulated that exposure to GBV can result in trauma and stress-related physiological processes that can affect metabolism and appetite, the development of eating disorders, coping mechanisms that influence nutrition and eating behaviors, and weight gain [39–42]. Specifically, evidence indicates that disordered eating patterns and overweight/obesity among survivors of CSA and IPV/dating violence emerge in adolescence and have enduring effects in adulthood [43, 44]. Also, forms of GBV affecting girls, such as child marriage, the preferential feeding of men and boys, and controlling behaviors, may directly and indirectly impact determinants of nutrition—namely, access to nutrient-rich foods and nutrition-related services—potentially contributing to malnutrition in adolescence [45–48]. That we are aware, there is no existing evidence synthesis focused on summarizing the current body of knowledge surrounding the childhood and adolescence nutritional consequences of GBV.

### Purpose

The purpose of the present research is to review and synthesize peer-reviewed, quantitative evidence describing the associations between GBV against girls and indicators of girls’ nutrition outcomes during childhood and adolescence. We focus on GBV perpetrated against girls given the burden of GBV among girls and to delineate sex-specific mechanisms reported among girls. We conducted a rapid evidence assessment (REA) to describe the existing evidence-base, delineate potential causal mechanisms and pathways in the available data, and identify key methodological considerations for future empirical and theoretical research endeavors.

## Methods

The present review is part of a larger REA commissioned by the United Nations Children’s Fund (UNICEF). The larger REA had two research objectives: (1) Review and synthesize quantitative evidence describing the associations between GBV against girls and indicators of girls’ nutrition outcomes (Direct GBV exposure) and (2) Review and synthesize the quantitative evidence describing the associations between IPV against a maternal caregiver and children’s nutrition outcomes, with a particular focus on humanitarian contexts (Indirect GBV exposure). The present paper is focused specifically on the first objective of the larger REA, pertaining to the direct pathway. A separate publication details the indirect pathway (Authors, under review). There is no published review protocol. However, the protocol for the larger REA is presented in Appendix B of S2 Appendix.

We adapted the Preferred Reporting Items for Systematic Reviews and Meta Analysis standards (PRISMA) [49] for the purposes of conducting a REA. REAs adapt systematic review methodology to synthesize a body of literature, ensuring rapid completion of the review and applicability to policy and programmatic needs in a short timeframe [50]. The following changes to systematic review methodology were made: (i) title/ abstract screening was conducted by one of three reviewers and (ii) data extraction and quality assessment were conducted by a one or two team members per article, depending on whether the article warranted discussion. Furthermore, while inclusion and exclusion criteria were determined a-priori, the team iteratively refined the criteria based on the volume of sources and the overall research goal. We conducted the REA in 7 stages, which we describe below: (1) Preliminary scoping of the literature to clarify the research question and search strategy, (2) Search strategy execution, (3) Title and abstract screening, (4) Full text review, (5) Quality Assessment, (6) Data extraction, and (7) Synthesis.

As a preliminary step, we conducted a preliminary scoping of existing reviews and literature on the associations between girls’ direct exposure to GBV during childhood and nutrition outcomes. We searched Google Scholar, Cochrane Reviews and PubMed for reviews concerning direct childhood exposures to GBV and subsequent nutrition-related outcomes. Selected reviews and literature were examined to elucidate specific direct pathways, select forms of GBV to include in the review, and to help operationalize database search terms.

Our preliminary scoping review indicated a general lack of robust research synthesis detailing the effect of girls’ exposure to GBV on adverse nutrition outcomes during childhood and adolescence. In our preliminary scoping review, we identified the following forms of GBV experienced during childhood that are potentially related to later nutrition deficits among females: preferential feeding of boys, gendered child abuse (i.e., CSA), child marriage, and IPV/dating violence.

### Eligibility criteria

Eligibility criteria were defined *a-priori* and then were iteratively refined to better reflect the existing body of literature and address the research question. Inclusion and exclusion criteria are displayed in Table 1. Two adjustments to the a-priori eligibility criteria were made. First, we originally wanted to focus on empirical evidence conducted in humanitarian settings. However, due to the limited number studies detailing girls’ direct exposure to GBV and nutrition related outcomes in humanitarian settings, we expanded the eligibility criteria to include studies conducted in any setting or country.

**Table 1:** Inclusion criteria.

	Inclusion Criteria	Exclusion Criteria
**Study Type**	Peer-reviewed literature	Grey literature, dissertations, presentations, book chapters, single case study
**Study Design**	Utilized any quantitative methodology; mixed methods studies were included if quantitative findings were separately reported	Fully qualitative methods and results
**Publication Date**	Published after the year 2000	Published before the year 2000
**Language**	Published in English or Spanish	Published in languages other than English or Spanish
**Exposure**	Included the following direct exposures for girls under the age of 20: child marriage, early marriage, forced marriage, preferential feeding of men/boys, sexual violence, any form of IPV/dating violence (physical, sexual, psychological, or controlling behaviours).	Did not include the forms of GBV affecting girls, as identified in the inclusion criteria, as exposure or primary interest variables. Did not present a bivariate or multivariate measure of association that could be attributed to the forms of GBV identified in the inclusion criteria (i.e., if multiple forms of violence against girls were aggregated or if GBV was merely controlled for and no measure of association was provided). Any measures of women/girls’ empowerment or empowerment without exposure to gender-based violence.^a^
**Outcomes**	Iron deficiency anemia; Gestational diabetes (if occurring in adolescent mothers); Below ten percentile weight; Below ten percentile height; Length for age; Risk for stunting and/or acute wasting; Failure to thrive; Nutrient deficiencies; Iron deficiency anemia; vitamin D deficiency/rickets; protein-energy malnutrition; Risk for overweight/ obesity; Less healthy dietary practice; underweight/ low body mass index; Low mid arm circumference; minimal dietary diversity, minimal meal frequency, amenorrhea, and minimum acceptable diet.	Did not measure or include the relevant direct pathway nutrition outcomes, as identified in the inclusion criteria. Studies that included mental health outcomes related to nutrition (i.e., body dysmorphia, eating disorders) but did not also measure the nutrition indicators of interest were excluded. Included nutrition-related outcomes as covariates without presenting a measure of association.
**Population of interest**	Girls (under 20 years) with respect to the GBV exposure(s) and the malnutrition outcome(s). Studies that included malnutrition outcomes that were measured between 18–20 years were also included ^a^ Mixed gender samples were included only if results were stratified by gender and female measures of association were presented.	Sample was exclusive to males. Sample included both males and females but did not disaggregate relevant results by gender/sex. Sample was exclusive to nutrition-related outcomes measured in adulthood.
**Setting**	All settings: high-middle-and-low-income countries as well as humanitarian settings and countries. ^a^	Did not restrict by setting

^a^ Denotes exclusion criteria that were determined iteratively at the full text review stage

Second, we originally conceptualized the population of interest to be females up to the age of 18. However, we noted that sensitive periods of overweight development following sexual abuse or IPV occur between adolescence and young adulthood. In our title and abstract screening of the direct pathway studies, we found several longitudinal studies also measured nutrition outcomes into adulthood. In an effort not to erroneously exclude samples of children followed into adulthood, and to be consistent with the WHO definition of adolescence which extends through age 19 years, we included nutrition outcomes also measured among girls who were up to 20 years old. Although many sources recruited samples that contained both males and females, all sources included in this REA provided sex-stratified measures of association. We used these to elucidate the association between exposure to gender-based violence and nutrition indicators in females during later childhood and adolescence.

### Search protocol and study selection

We conducted a structured database search in Medline, Embase, and Global Health using the Ovid Medline platform in July 2021 and in November 2022 (to capture newly published research). Boolean operators were used to best account for keyword and MeSH term variations. No filters, limits, or restrictions were applied to the search. Sources were exported to Covidence, a web-based software platform designed to streamline screening and full text review for systematic reviews. The S1 Appendix presents the full search strategy for each database.

Screening occurred in two stages: (i) title and abstract; and (ii) full text review. Titles/abstracts were each screened by one reviewer. Prior to conducting single screening, the team practiced screening on a sub-sample of titles/abstracts (n = 400) to ascertain and resolve any differences in interpretation regarding how to apply the inclusion and exclusion criteria. During the title/abstract screening phase, when reviewers were uncertain on how to apply the inclusion/exclusion criteria in relation to a particular source, the team discussed and reached consensus. During the full text screening, sources were assessed vis-à-vis the inclusion/exclusion criteria by two independent reviewers. Discordance between the reviewers was adjudicated by a third, independent reviewer and when necessary, further discussed with the wider team to reach consensus.

### Quality assessment and data extraction

Given the diverse quantitative study designs included, no single quality assessment tool was deemed appropriate. Accordingly, the team combined relevant quality assessment questions from the following quality assessment tools: National Institutes of Health (NIH) [51], Mixed Methods Assessment Tool (MMAT) [52], Appraisal Tool for Cross Sectional Studies (AXIS) [53], Newcastle-Ottawa Scale (NOS) [54], and the National Institutes of Health assessment tool for observational cohort and cross sectional studies [55]. The items included in the quality assessment are presented in Table 2.

**Table 2:** Quality assessment domains.

Source	Quality Assessment Item
NIH	Was the research question or objective in this paper clearly stated?
NOS	Was the sample: (1) Truly representative of the average in the target population (all subjects or random sampling) (2) Somewhat representative of the average in the target population. (non-random sampling) (3) Selected group (i.e., clinic-based, sampling in hospital) (4) No description of the sampling strategy
AXIS	Was the target/reference population clearly defined? (Is it clear who the research was about?)
MMAT	Is the sampling strategy relevant to address the research question?
NOS	Non-Respondents and Response Rate: (1) Comparability between respondents and non-respondents’ characteristics is established, and the response rate is satisfactory (2) The response rate is unsatisfactory, or the comparability between respondents and non-respondents is unsatisfactory (3) No description of the response rate or the characteristics of the responders and the non-responders.
n/a^a^	Was the violence exposure measured rigorously?
n/a^a^	Was the nutrition outcome measured rigorously?
NIH	Were key potential confounding variables measured and adjusted statistically for their impact on the relationship between exposure(s) and outcome(s)?
NOS	(1) The statistical test used to analyze the data is clearly described and appropriate, and the measurement of the association is presented, including confidence intervals and the probability level (p value). (2) The statistical test is not appropriate, not described, or incomplete.

^a^ Designates quality assessment questions that were created in response to the research goals, rather than adapted from an existing tool

The team designed a data extraction template in accordance with the research goals. Specifically, data were extracted according to general study characteristics, elements of the study design and sampling, violence exposure measurement, nutrition outcome measurement, and summary of results. The data extraction template was pilot tested for further refinement. Team members extracted information independently and extraction was reviewed by a senior member of the research team.

### Synthesis

Once data extraction and the quality assessment were completed, the team reviewed the evidence base for patterns. Overarching patterns and findings with respect to study design and sampling, GBV and nutrition indicator measurement methods, overall direction of findings, and mechanisms/pathways connecting exposure to outcome were described and summarized into tables. In particular, we grouped studies by mechanism/pathway studied and synthesized overall results according to mechanism/pathway. Quality assessment measures were summarized using aggregate descriptive statistics (count frequencies and percentages). See S2 Appendix for disaggregated quality assessment results.

## Results

**Fig 1:**
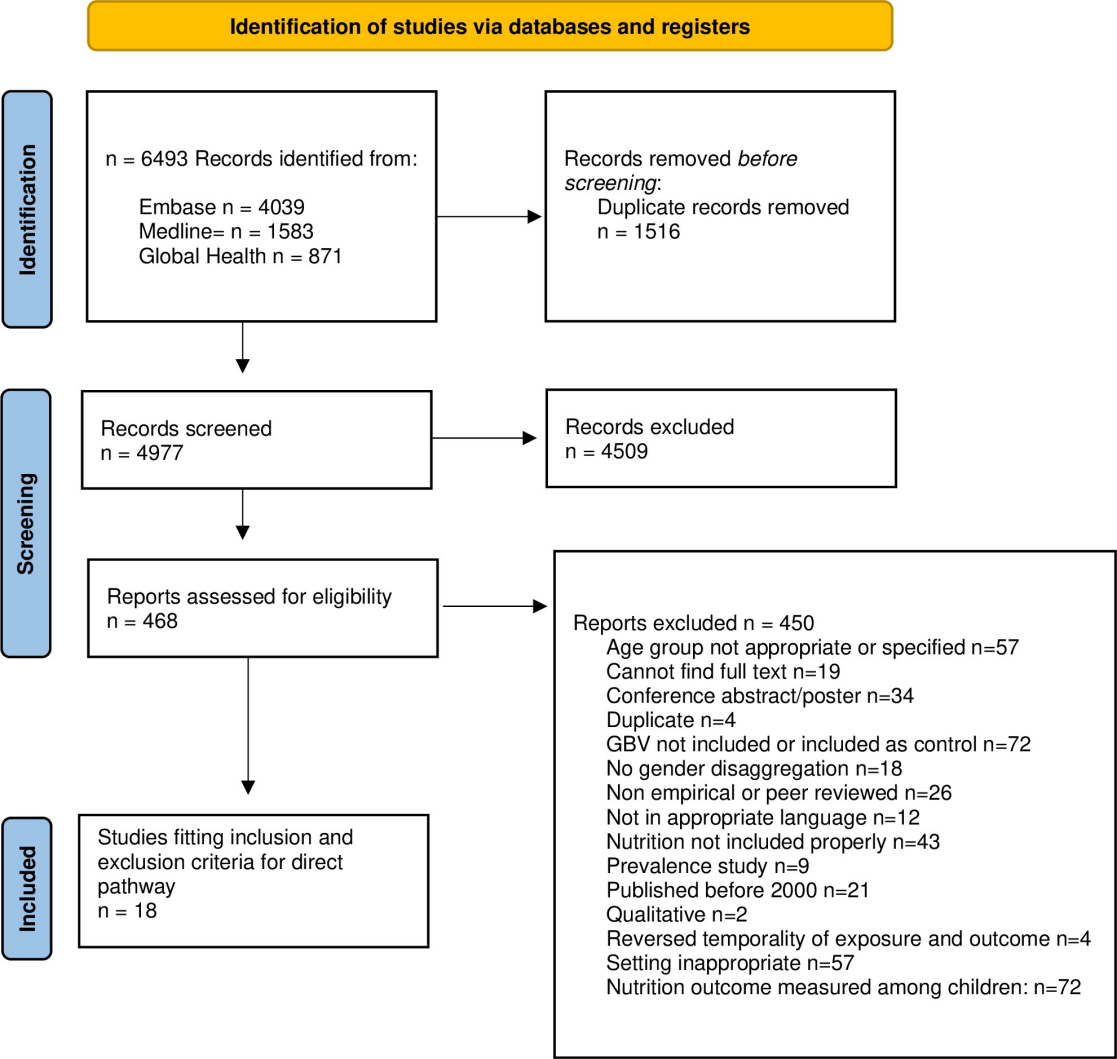
PRISMA flow chart.

The PRISMA flow diagram is presented in Fig 1. After executing the database search strategies, 6493 sources were imported to Covidence and 1516 duplicates were removed, yielding 4977 citations for screening. During title and abstract screening, 4461 sources were excluded, and 468 full texts were assessed for eligibility. During full text review, 450 sources were excluded for various reasons outlined in Fig 1. In total, only 18 represent the direct pathway and are included in this paper.

### Study characteristics

Table 3 displays general characteristics for the 18 studies examining pathways between girls’ GBV exposure and indicators of nutrition outcomes during childhood and adolescence. Although more than half of studies were conducted in high-income settings, several studies reported outcomes in low-and-middle income countries. The specific countries included were Ethiopia (n = 1), Nepal (n = 1), Brazil (n = 2), Mexico (n = 1), as well as United States (n = 8), England (n = 2), and the Netherlands (n = 2), with one study combining data from the United Kingdom and Brazil (n = 1).

**Table 3:** Study characteristics.

Article	Research question	Country, WHO regional classification, Humanitarian country (Yes/No)	Study Design	Secondary Data Use: Yes/ No If yes, which dataset?
Anderson (2015)	Examine the relationship between sexual assault and overweight as well as their association with suicide risk, among adolescents	United States, AMR, No	Cross sectional	Yes—2009 and 2011 National Youth Risk Behavior Surveys
Clark (2014)	Test whether exposure to dating violence is associated with higher BMI across adolescence to young adulthood, and whether previous exposure to child maltreatment and gender modify associations	United States, AMR, No	Longitudinal	Yes- National Longitudinal Study of Adolescent Health
Denholm (2013)	Establish whether household dysfunction, child abuse, and neglect were associated with height, growth, and adult leg length, at what age any associations were apparent, and whether relationships persisted in adulthood	England, EUR, No	Prospective cohort	Yes—1958 British Birth Cohort
Elsenburg (2022)	Asses the relationship between childhood abuse and BMI measured in young adulthood, taking into consideration mediation by major depressive disorder and generalized anxiety disorder.	Denmark, EUR, No	Prospective cohort	Yes- Tracking Adolescents’ Individual Lives Survey
Li (2021)	Test whether attenuation of the hypo-thalamic-pituitary-adrenal axis explains the elevated risk of obesity among sexually abused females.	United States, AMR, No	Longitudinal	Yes- Female Growth and Development Study
Souza Marques (2021)	Explore the association between interpersonal violence and inadequate nutrition among Brazilian adolescents	Brazil, AMR, Yes	Cross sectional	Yes—Brazilian National Survey of School Health
Martin Martin (2010)	To examine the nutritional impact of past physical and sexual abuse on children less than 12 years old.	Mexico, AMR, Yes	Cross sectional	No
Noll (2007)	Track BMI across development using a prospective study of females who experienced reported child sexual abuse	United States, AMR, No	Longitudinal	No
Peckins (2019)	Examine the association between maltreatment types and BMI trajectories over time, and whether BMI changes were conditional on boys’ and girls’ cortisol reactivity	USA, AMR, No	Longitudinal	No
Schiff (2021)	To assess the relationship between ACEs and obesity status during childhood.	USA, AMR, No	Longitudinal	Yes- National Survey of Child and Adolescent Wellbeing.
Schneiderman (2012)	(1) To compare BMI (overweight and obese) among maltreated and non-maltreated adolescents, (2) Determine correlates of high BMI, (3) Identify type of maltreatment related to high BMI	USA, AMR, No	Cross sectional	No
Schneiderman (2014)	Compare the association between childhood maltreatment and BMI trajectory with respect to two comparator groups	USA, AMR, No	Longitudinal	No
Soares (2018)	Assess the association between exposure to adverse childhood experiences and adiposity (BMI, waist circumference, fat mass index, android fat percentage) in adolescents in Brazil and the United Kingdom	(1) United Kingdom, EUR, No (2) Brazil, AMR Yes	Cross-cohort comparison	Yes—(1) Avon Longitudinal Study of Parents and Children (2) 1993 Pelotas Birth Cohort
Soares (2021)	Examine associations between childhood abuse and cardiometabolic health (including BMI) in young adulthood.	United Kingdom, EUR, No	Prospective cohort	Yes- Avon Longitudinal Study of Parents and Children
Souza Marques (2022)	Investigate the relationship between interpersonal violence (physical and sexual abuse) and nutritional status (BMI categories) among adolescents.	Brazil, AMR, Yes	Cross sectional	Yes- 2015 Brazilian National Survey of School Health
Tiruneh (2021)	Assess whether early childbearing and marriage were associated with anemia among adolescent girls	Ethiopia, AFR Yes	Cross sectional	Yes–Demographic and Health Survey (DHS)
Veldwijk (2012)	(1) Assess the prevalence of physical, sexual, and mental abuse of adolescents (2) Analyze associations between abuse and BMI status among adolescents	Netherlands, EMR No	Panel (2 cross sectional surveys in 2003 and 2007)	Yes—E-MOVO
Wells (2022)	Test if earlier first pregnancy and earlier marriage are associated with poor nutritional status of mothers, in a dose response relationship. Test whether the associations of nutritional status and maternal age and marriage age are independent of one another.	Nepal, SEAR, Yes	Cluster Randomized Controlled Trial	Yes- Low Birth Weight South Asia Trial

Most sources sought to quantify associations between specific forms of sexual violence affecting children, including girls, and indicators of subsequent nutrition status in childhood and adolescence. Some studies conducted analyses that permit more nuanced understandings of causal mechanisms including use of community-based comparator groups [56–59]; moderation by cortisol reactivity [57]; moderation by HPA axis attenuation [41]; interaction of caregiver abuse and IPV/dating violence [60]; interaction of child marriage and age at first pregnancy [61]; and stratification by age [62] and developmental period [56].

### Quality assessment

In Table 4 we present the quality assessment results. Key gaps in quality were due to high attrition/non-response, poor measurement of GBV, and limited external validity/generalizability due to the sampling of selected groups. All studies (100%) had clearly stated research questions and objectives. Studies varied in terms of external validity and representativeness, with 55.6% of studies being truly representative of the target population and 38.9% representing a selected group, for example, cases of child abuse reported to Child Protective Services. Less than half of studies (38.9%) achieved satisfactory response rates (meaning less than 10% attrition/non-response) and/or comparability between respondents and non-respondents. The measurement of girls’ exposure to gender-based violence was deemed rigorous in half of the included studies (50.0%) and the measurement of childhood nutrition-related outcomes was appropriately rigorous in 83.3% of the included studies. Rigorous measurement of GBV and nutrition was assessed using a variety of indicators including, whether the variables were clearly defined, accurately measured and applied consistently, measurements were justified and appropriate for answering the research question, measurements reflected what they are supposed to measure, measures were collected using validated and reliability tested tools/instruments/questions, variables were measured using ‘gold standard’ tools, and questionnaires were pre-tested prior to data collection. Two thirds (66.7%) of studies adequately controlled for confounding variables. Lastly, 22.2% of studies did not properly conduct or report on their statistical analysis.

**Table 4:** Quality assessment.

Quality Assessment item	Possible responses	Number of articles meeting criteria	Percent (Calculated from a total of 18 studies)
**Introduction**			
Was the research question or objective in this paper clearly stated?	Yes	18	100.0%
**Methods**			
Representativeness of the sample	Truly representative of the average in the target population (all subjects or random sampling)	10	55.6%
	Selected group (i.e. clinic-based sampling in hospital)	7	38.9%
Was the target/reference population clearly defined?	Yes	12	66.70%
Is the sampling strategy relevant to address the research question?	Yes	12	66.70%
Non-Respondents	(1) Comparability between respondents and non-respondents is established, and the response rate is satisfactory	7	38.9%
	(2) The response rate is unsatisfactory or the comparability between respondents and non-respondents is unsatisfactory	10	55.6%
	(3) No description of the response rate or the characteristics of the responders and the non-responders.	1	5.6%
Is the violence exposure measured rigorously?	Yes	9	50.0%
**Results**			
Is the nutrition outcome measured rigorously?	Yes	15	83.3%
Were key potential confounding variables measured and adjusted statistically for their impact on the relationship between exposure(s) and outcome(s)?	Yes	12	66.7%
**Statistical test**	The statistical test used to analyze the data is clearly described and appropriate and the measurement of the association is presented including confidence intervals and the probability level (p value)	14	77.8%
	The statistical test is not appropriate not described or incomplete.	4	22.2%

### Sampling

Table 5 presents participant recruitment and sample details. Gender-stratified samples ranged from under 100 females [56] to over 26,000 [63]. The range of sample sizes is a reflection of the study designs utilized; studies with smaller sample sizes focused on newly reported cases of CSA obtained from Child Protective Services [41, 56–59], or other similar organizations such as a clinic for victims of child abuse [64], and larger sample sizes pertained to secondary data analyses of nationally representative and/or longitudinal datasets of child and adolescent health [60, 62, 63, 65–71]. Sampling of CSA cases from Child Protective Services is a valid method for capturing this form of GBV given that cases of genital contact and/or perpetration by family members are investigated and substantiated according to national laws. In such studies, the survivor children and a non-abusing caregiver(s) are referred to the study within a specific time frame (i.e., 6 months) of the initial abuse disclosure. However, sampling of CSA cases from Child Protective Services may not represent the entire population of CSA cases given that only a proportion of children who experience this form of abuse formally enter the child protection system. Socio-economic status and racialization experiences (as well as other social determinants of health) may differ between CSA cases that formally enter protection systems and cases that are unreported, thereby potentially introducing sampling bias and limiting generalizability.

**Table 5:** Participant recruitment and sampling.

Article	Sample size (n); Inclusion criteria	Participant recruitment	Probability sample	Representativeness of the sample
Anderson (2015)	n = 31,540 adolescents (50.4% female and 49.6% male); not included	Three-stage cluster sample design	Yes	Truly representative of the average in the target population (all subjects or random sampling)
Clark (2014)	n = 9295 adolescents; Adolescents between grade 7 and 12 in the US, between the 1994–1995 school year. Analysis includes respondents who participated in all 4 waves of the survey, had non missing sampling weights, reported at least on relationship with respect to dating violence	Representative selection of US-based middle and high schools, and sampling of students within schools using multistage sampling	Yes	Truly representative of the average in the target population (all subjects or random sampling)
Denholm (2013)	n = 17,638 births from a birth cohort; Born in England, Wales, or Scotland during one week in Match of 1958. Children who immigrated in England and were born in the same week were enrolled during the childhood surveys.	Enrolled all live births in England, Wales, and Scotland born in March 1958 and immigrants born in same time. These children were followed up prospectively at the ages of 7, 11, 16, 23, 33, 42, 45, and 50 years	Yes	Truly representative of the average in the target population (all subjects or random sampling)
Elsenburg (2022)	N = 3486 births between 1989–1990 in selected areas in northern Netherlands. Included participants had to have parental consent, be without physical illness or cognitive disability, and had parents who could communicate in Cutch, Turkish, or Moroccan.	Children born between October 1989 and September 1990 from three municipalities located in the north Netherlands were recruited and followed up until April 2023-November 2013.	Yes	Truly representative of the average in the target population (all subjects or random sampling)
Li (2021)	n = 82 girls with family-member perpetrated CSA that was substantiated by Child Protective Services from Washington DC who were, 6–16 years old, enrolled within 6 months of reporting the abuse, and had a non-per perpetrating caregiver who could also participate. n = 84 non-abused comparator females were demographically matched (age, race, ethnicity, zip codes, socio-economic status, family constellation, non-sexual traumatic events) to the cases of sexual abuse.	Child Protective Services from Washington DC referred participants to the study between 1987–1989. Comparator group was recruited via newspapers and posters in the same neighborhoods of the sexually abused cases.	No	Selected group (i.e. clinic-based sampling in hospital)
Martin Martin 2010	n = 178 total children, 108 were girls; 41 with exposure to physical abuse, 65 with exposure to sexual abuse; children followed at a clinic for abused children from March 1994 –Sept 2005	This data is from a clinical chart review, no recruitment process	No	Selected group (i.e. clinic-based sampling in hospital)
Noll (2007)	n = 84 females who experienced abuse and 102 comparator participants; Females who experienced CSA, perpetrated by family members, who were referred by Child Protection Specialists in Washington DC. Had to be 6 to 16 years old, participated within 6 months of violence disclosure, substantiated sexual abuse, perpetrated by family member, participation of caregiver/family member who did not abuse the child	Child Protective Services in Washington, DC referred female victims of CSA. The victims were then compared to female participants recruited from the community and neighborhoods as the participants and were of similar ages and demographics. Female participants in the comparator group that had sexual abuse history were not included	No	Selected group (i.e. clinic-based sampling in hospital)
Peckins (2019)	n = 454 adolescents (242 males and 212 females); Adolescents considered for inclusion if they received a referral for maltreatment from the LA County Department of Children and Family Services within past month, were 9–13 years old, were Black, Latino, or white, resided within selected zip codes in urban Los Angeles (LA).	The participants who were exposed to maltreatment were referred by the LA County Department of Children and Family Services. Participants were contacted via post card and phone call. The comparison group was created by consulting school lists of children 9–12 from the selected zip codes of the maltreatment sample. Parents/caregivers of the children were contacted via postcard and phone call.	No	Selected group (i.e. clinic-based sampling in hospital)
Schiff (2021)	N = 3170 participants, tracked from birth to 14 years of age	Two-stage stratified sampling design, with 8 sampling strata among 8 states with the highest child welfare caseloads and 1 sampling strata of the remaining US. Representative of national population of children up to 17 years in families that were investigated for maltreatment.	Yes	Truly representative of the average in the target population (all subjects or random sampling)
Schneiderman (2012)	n = 303 (151 girls) in the maltreated group and 151 in the comparison group, Cases of childhood maltreatment referred and investigated by the LA County Department of Children and Family Services within the past 60 days, 9–12 years of age, Black, Latino or white, resided within specific zip codes in LA	The participants who were exposed to maltreatment were referred by the LA Count Department of Children and Family Services. Participants were contacted via post card and phone call. The comparison group was created by consulting a list obtained from a marketing firm to recruit children 9–12 from the selected zip codes of the maltreatment sample. Parents/caregivers of the children were contacted via postcard and phone call.	No	Selected group (i.e. clinic-based sampling in hospital)
Schneiderman (2014)	n = 303 (151 girls) in the maltreated group and n = 151 in the comparison group, Cases of childhood maltreatment referred and investigated by the LA County Department of Children and Family Services within the past 30 days, 9–12 years of age, Black, Latino, or white, resided within specific zip codes in LA	The participants who were exposed to maltreatment were referred by the LA Count Department of Children and Family Services. Participants were contacted via post card and phone call. The comparison group was created by consulting a list obtained from a marketing firm to recruit children 9–12 from the selected zip codes of the maltreatment sample. Parents/caregivers of the children were contacted via postcard and phone call.	No	Selected group (i.e. clinic-based sampling in hospital)
Soares (2018)	(1) UK birth cohort: n = 4,444 adolescents (2) Brazilian birth cohort: n = 3,924 adolescents; (1) UK birth cohort: pregnant women who delivered in Avon, UK between April 1, 1991, and December 31, 1992; live birth (2) Brazilian birth cohort: all live children born in urban areas of Pelotas, Brazil between Jan 1, 1993, and December 31, 1993	(1) Avon Longitudinal Study of Parents and Children: Pregnant women who lived in the Avon area, UK who delivered between April 1, 1991, and December 31, 1992, were recruited into the study. Follow up among the mothers/partners/children followed up over time. (2) 1993 Pelotas Birth Cohort: All children born alive within hospitals located in urban areas of Pelotas, Brazil between January 1, 1993, and December 31, 1993.	Yes	Truly representative of the average in the target population (all subjects or random sampling)
Soares (2021)	UK birth cohort: n = 3223 adolescents included in present analysis, n = 2143 females. The birth cohort included pregnant women who delivered in Avon, UK between April 1, 1991, and December 31, 1992. Having a live birth was necessary.	(1) Avon Longitudinal Study of Parents and Children: Pregnant women who lived in the Avon area, UK who delivered between April 1, 1991, and December 31, 1992, were recruited into the study. Follow up among the mothers/partners/children followed up over time.	Yes	Truly representative of the average in the target population (all subjects or random sampling)
Souza Marques (2021)	n = 11,850 adolescents; Children older than 13 years old	Multi-stage cluster sampling of schools, classrooms, and children within the classrooms selected.	Yes	Truly representative of the average in the target population (all subjects or random sampling)
Souza Marques (2022)	n = 2317 adolescent girls older than 13 years. Sampling included adolescents in elementary school (grades 6–9) and between the first and third year of high school. Analysis was restricted to children over 13 years.	Multi-stage cluster sampling	Yes	Truly representative of the average in the target population (all subjects or random sampling)
Tiruneh (2021)	n = 3172 girls; adolescent girls	As per DHS nationally representative sampling: two stage cluster sampling design of randomly selected communities and households.	Yes	Truly representative of the average in the target population (all subjects or random sampling)
Veldwijk (2012)	n = 51,865 adolescents (26,529 girls); Second and fourth grade secondary students in Eastern Netherlands.	Second and fourth grade secondary students in the Netherlands, that were located in the catchment area of 6 selected Community Health Services in 2003 and 2007. The survey was administered in-class using the internet.	Yes	Selected group (i.e. clinic-based sampling in hospital)
Wells (2022)	n = 4071 first time mothers with complete data and who were married after 10 years and had their first pregnancy before 30 years.	The cluster randomized controlled trial was conducted in rural lowland Nepal across 80 geographical clusters. 20,090 pregnant women were recruited into four possible interventions, that aimed to improve offspring birthweight.	No	Not representative

The majority of studies included samples of adolescent participants or samples in which participants were adolescents/young adults at final data collection; although Martin-Martin et al. [64] specifically focused on children ages 0–11 years. Participant recruitment strategies varied across the sources and included both probability (n = 11) and non-probability samples (n = 7). Two predominant study designs were utilized: (i) longitudinal—including panel, prospective cohort, and cross cohort comparison designs—(n = 12) and (ii) cross sectional (n = 6). Thirteen studies conducted secondary analysis on existing datasets.

### Violence exposures

The following forms of GBV were measured: child marriage [61, 68], sexual assault and rape [63, 65, 66], CSA perpetrated by parents/caregivers [41, 56–59, 62, 64, 67, 69–71], and dating violence/IPV [60] (Table 6). Some studies did not use validated survey instruments to measure GBV [63, 65–67, 70, 71], and in some cases relied on a single question to assess girls’ GBV exposure [63, 66, 67]. Clark et al., (2014) [60] employed the Conflict and Tactics Scale, which is a widely used and validated instrument. Martin-Martin et al., (2010) [64] used clinical records to document exposure to sexual abuse.

**Table 6:** Violence exposure.

Study	Exposure Type	Form of violence	Perpetrator Identity	Timeframe of the Violence Exposure	Exposure Measurement
Anderson (2015)	Sexual Assault	Sexual	Any perpetrator	Lifetime	Non-validated survey
Clark (2014)	IPV	Physical and Sexual	Current or previous intimate partner	Wave II: Previous 18 months; Wave III: Since summer of 1995; Wave IV: Since current relationship	Conflict Tactics Scale
Denholm (2013)	Domestic violence; sexual abuse	Physical and Emotional/ Psychological/ Verbal and Sexual; Financial	Male or female family member	During childhood by the age of 16	Other, Abuse and witnessing abuse measured at 45 years using a questionnaire used in a previous project, which adapted questions from existing instruments.
Elsenburg (2022)	Childhood abuse	Sexual	Adult family member, family acquaintance, stranger	CSA victimization prior to the age of 16.	Non-validated survey: Questionnaire developed by study team, consisting of 5 questions on sexual abuse.
Li (2021)	CSA	Sexual	Male or female family member	Violence disclosure up to 6 months prior to participating in the survey	Other, Referral from Child Protective Services
Martin Martin (2010)	Non-partner sexual assault, suspected sexual and physical abuse based on clinical indicators	Sexual	Male or female family member; intimate partner	Lifetime	Review of records (medical/police),
Noll (2007)	CSA	Sexual	Male or female family member	Violence disclosure up to 6 months prior to participating in the survey	Other, Referral from Child Protective Services
Peckins (2019)	CSA	Sexual	Male or female family member	Referral from LA County Department of Children and Family Services within past 30 days	Combination, (1) review of juvenile court case records AND (2) referral from LA County Department of Children and Family Services
Schiff (2021)	Childhood abuse	Sexual	Adult or older child, including anyone who was a member of the family.	Past year victimization	Any forced sexual contact recorded using the parent-child conflict tactics scale.
Schneiderman (2012)	CSA	Sexual	Male or female family member	Referral from LA County Department of Children and Family Services within past 60 days	Combination, (1) review of child welfare case records AND (2) referral from LA County Department of Children and Family Services
Schneiderman (2014)	CSA	Sexual	Male or female family member	Referral from LA County Department of Children and Family Services within past 30 days	Combination, (1) review of child welfare case records AND (2) referral from LA County Department of Children and Family Services
Soares (2018)	Sexual assault/ CSA; domestic violence	Physical and Sexual	Male or female family member	Lifetime (Brazil) and since last follow up survey (UK)	Non-validated survey, Survey questions included as part of the 1993 Pelotas Cohort and the Avon UK birth cohort
Soares (2021)	CSA	Sexual	Adult or older child	Before 11 years, between 11 and 17 years. Asked retrospectively at 22 years.	Non-validated survey; two survey questions inquiring about sexual rouching and attempted forced/forced sexual activity.
Souza Marques (2021)	Domestic violence; sexual assault	Physical and Sexual	Any perpetrator	Past month	Non-validated survey, Survey questions from the Brazilian National Survey of School Health. The questionnaire was based on other studies involving school-aged youth. The questionnaire was also adapted and tested according to the Brazilian context.
Souza Marques (2022)	Rape	Sexual	Any perpetrator	Past 30 days (during adolescence)	Single survey question: “Have you ever been forced to have sexual intercourse?”
Tiruneh (2021)	Child marriage	N/A	N/A	Lifetime	Demographic and Health Survey Questions
Veldwijk (2012)	Sexual violence	Sexual	Any perpetrator	Lifetime	Non-validated survey, Lifetime sexual violence by any perpetrator was assessed using one single survey question
Wells (2022)	Child marriage (and the related exposure of age at first pregnancy)	N/A	N/A	Lifetime	Survey questions

### Nutrition indicators

Nutrition indicators relied on anthropometric measures that were either observed or self-reported (Table 7). Body mass index (BMI) was the most used nutrition indicator included in the studies of older children and adolescents. In addition, other nutrition indicators that were assessed included height for age, weight for height, height for younger children, leg length, waist circumference, fat mass index, android fat percentage, mid-upper arm circumference (MUAC), and anemia among the older children and adolescents. Height and weight were assessed using either anthropometric measurements [41, 56–61, 64, 66, 67, 69–71] or self-reported height and weight (which may be affected by recall error or bias based on context dependent body ideals) [63, 69, 72]. Either weight for height or BMI were calculated as appropriate according to participants’ ages and operationalized for analysis using different methods [73, 74].

**Table 7:** Nutrition outcomes.

Article	Outcome	Measurement approach	Definition	Developmental time frame	UNICEF developmental time frames
Anderson (2015)	Waist circumference	Anthropometric measurements	Waist circumference was measured during clinical visits, at the middle point of the lower ribs and the iliac crest (UK cohort) and at the narrowest point of the waist (Brazil cohort)	Adolescence (puberty up to 19 years)	10–19 years
	Fat mass index and android fat percentage	Anthropometric measurements	Fat mass index: using dual-energy x-ray absorptiometry, total fat mass was divided by height Android fat percentage: proportion of android fat mass to total body fat mass	Adolescence (puberty up to 19 years)	10–19 years
	Overweight	Self-report using validated instrument	Participants self-reported their height and weight. BMI percentile scores were then calculated based on CDC growth charts (BMI-for-age). Dichotomized as: healthy weigh <85 percentile and overweight (≥ 85 percentile).	Adolescence (puberty up to 19 years)	10–19 years
Clark (2014)	BMI	Anthropometric measurements	At waves II, III, and IV BMI was calculated based on height and weight measurements that were also collected	Adolescence (puberty up to 19 years)	10–19 years
Denholm (2013)	Height	Anthropometric measurements	Trained medical personnel measured height and leg height at 7, 11, 16, 33, and 45 years. Leg height obtained by subtracting sitting height from standing height. Continuous variable, measured in cm.	School age (5–11 years); Adolescence (puberty up to 19 years)	5–9 years,10–19 years
Elsenburg (2022)	BMI	Anthropometric measurements	Trained research assistants collected measures at waves 4 (18–21 years) and wave 5 (21–23 years) using calibrated scales for weight and stadiometers/measuring tapes for height. Participants were dressed lightly.	Adolescence (puberty up to 19 years)	10–19 years
Li (2013)	BMI, Adult obesity status	Anthropometric measurements	Trained study staff measured height and weight among participants in clothing without shoes using calibrated Health-O-Meter balance bean scales. CDC guidelines for obesity were followed: age and sex specific BMI percentile of 95 or higher (≤19 years) and BMI cutoff score of 30km/m2 (>19 years). Measured were assessed at the mean ages of 11, 12, 13, 18, 19, and 24 years, which corresponded to 6 developmental time periods.	School age (5–11 years); Adolescence (puberty up to 19 years).	10–19 years
Martin Martin (2010)	Acute malnutrition, Overweight, Stunting	Review medical records, child abuse clinic, anthropometric measures	Weight for height (in children less than 60 months of age) using WHO cut points and Z score thresholds (>2SD); BMI using Z score thresholds in children 5–11 years; Underweight defined using weight for age in children less than 60 months using WHO criteria and z scores; Stunting using WHO criteria (height for age) and Z scores (>2SD)	Infant, toddlers, Pre-school (3 months—5 years); School age children (5–11 years)	0–23 months; 0–59 months; 5 to 9 years; 10–19 years
Noll (2007)	BMI and Obesity	Anthropometric measurements	Trained personnel measured height and weight of the participants using the calibrated Health-O-Meter balance mean scale. Obesity was defined according to CDC BMI z scores and dichotomized. ≥95th percentile if younger than 19 years of age and BMI ≥ 30 once participants reached age 20.	School age (5–11 years); Adolescence (puberty up to 19 years)	10–19 years, 5–9 years
Peckins (2019)	BMI	Anthropometric measurements	Graduate research assistants who received training measured height and weight using Health-o-meter scale, after removing shoes/bulky clothes. Repeated measures were taken and averages. BMI percentile scores were calculated.	School age (5–11 years); Adolescence (puberty up to 19 years)	10–19 years, 5–9 years
Schiff (2021)	Weight-for-age	Self-report	Caregivers indicated weight of the child participants. 95 percentile sex-specific weight-for-age was used to categorize child obesity, as per the CDC 2000 growth charts.	School age (5–11 years); Adolescence (puberty up to 19 years)	10–19 years
Schneiderman (2012)	BMI	Anthropometric measurements	Using mounted stadiometer, after removing shoes/bulky clothes. Repeated measures were taken and averages. BMI percentile scores were calculated. 4 weight groups were categorized (categorical variable) using CDC percentiles: (1) obese (≥95th); (2) overweight (≥85th to <95th); (3) normal weight (>5th to <85th); and (4) underweight (≤5th).	School age (5–11 years); Adolescence (puberty up to 19 years)	10–19 years, 5–9 years
Schneiderman (2014)	BMI	Anthropometric measurements	Using Healthometer scale, after removing shoes/bulky clothes. Repeated measures were taken and averages. BMI percentile scores were calculated.	School age (5–11 years); Adolescence (puberty up to 19 years)	10–19 years, 5–9 years
Soares (2018)	BMI	Anthropometric measurements	In both cohorts, height and weight were measured at a clinical visit.	Adolescence (puberty up to 19 years)	10–19 years
Soares (2021)	BMI	Anthropometric measures	Height and weight measured during clinic visits at 18 and 25 years.	Adolescence (puberty up to 19 years)	10–19 years
	Total cholesterol; plasma triglycerides; high density lipoprotein	Biomarkers	Enzymatic reagents for lipid determination using Lipid Research Clinics Protocol.	Adolescence (puberty up to 19 years)	10–19 years
	Low-density lipoprotein; cholesterol concentrations	Biomarkers	Friedewald equation	Adolescence (puberty up to 19 years)	10–19 years
Souza Marques (2021)	BMI	Anthropometric measurements	Weight was measured using an electronic and portable scale and height was recorded using a portable stadiometer. Measurements were recorded in duplicate to the first decimal. BMI classification was created by consulting the International Obesity Task Force. Ordinal variable: underweight, normal weight, excess body weight (overweight and obesity)	Adolescence (puberty up to 19 years)	10–19 years
Souza Marques (2022)	BMI	Anthropometric measurements	Weight and height were measured using a portable electronic scale and portable stadiometer. Measurements were performed in duplicate, with discrepancies resolved during third measure. BMI was operationalized according to the International Obesity Task Force (age and sex specific): underweight (BMI z-score of <−2), eutrophic (−2 ≥ BMI z-score ≤ +1), overweight (+1> BMI z-score ≤ + 2), and obese (BMI z-score> +2).	Adolescence (puberty up to 19 years)	10–19 years
Tiruneh (2021)	Iron deficiency anemia	Biomarkers	Home blood collection measuring hemoglobin levels. Cut offs for anemia were defined by the WHO: mild (10.0–10.9g/dl), moderate (7.0–9.9g/dl), and severe anemia (<7.0g/dl). Variable was dichotomized where in 0 indicated absence of anemia and 1 indicated presence of anemia.	Adolescence (puberty up to 19 years)	10–19 years
Veldwijk (2012)	BMI	Self-report using instrument not validated	Participants self-reported their own height and weight. Categorical variable with 4 levels was created: underweight, normal weight, overweight, or obesity. Underweight category was defined by Dutch reference population cut off and overweight/obesity defined by international gender/age specific cut offs.	Adolescence (puberty up to 19 years)	10–19 years
Wells (2022)	BMI	Anthropometric measurements	Various clinic staff measured height and weight using the Shorr boards and Tanita solar scales, respectively. Measured in early pregnancy (8–13 weeks) and at endpoint (within 25 months of delivery). Chronic energy deficiency (CED) was operationalized as BMI <18.5 kg^2^	Adolescence (puberty up to 19 years)	10–19 years
	Mid upper arm circumference (MUAC)	Anthropometric measurements	Using non-stretchable tape, various clinic staff measured MUAC at the endpoint (within 25 months of delivery).	Adolescence (puberty up to 19 years)	10–19 years

Height and leg length were assessed longitudinally throughout childhood and adolescence in Denholm et al., [62]. Multiple measures of adiposity were measured by Soares, et al., [67] using anthropometric measurements and dual-energy x-ray absorptiometry scans in adolescents. ‘Anemia’ during adolescence was measured in only one study using a point of care diagnostic lab test for hemoglobin and operationalized using WHO defined thresholds and presuming iron deficiency without documenting iron/ferritin levels [68].

### Synthesis of findings

The 18 included studies addressed three pathways describing associations and potential mechanisms between exposure to GBV during childhood/adolescence and nutrition indicators. Study results are displayed in Table 8. Overall, findings indicate that sexual abuse (for example, CSA, IPV, dating violence) experienced during childhood and adolescence was positively and significantly associated with elevated BMI, overweight, obesity, and adiposity, particularly when studied longitudinally. We found more limited evidence with respect to the relationship between child marriage and undernutrition (anemia, BMI, MUAC, and chronic energy deficiency). We outline the three pathways below, with a focus on summarizing results and describing important methodological implications.

**Table 8:** Results.

Pathway 1a: Exposure to sexual abuse by parents/caregivers during childhood and adolescence and later elevated BMI (i.e., overweight, obesity) or increased adiposity					
**Study**	**Adjusted Result(s)**	**Potential Confounders**	**Mediators**	**Moderators**	**Summary of findings/results**
Elsenburg (2022)	Exposure to CSA was associated with higher BMI at wave 4 (*β* = 0.97, 95%CI: 0.01,1.96), higher increase in BMI between wave 4–5 (B = 0.52, 95%CI: 0.04,1.01), and elevated BMI at wave 5 (*β* = 1.46, 95%CI: 0.36, 2.55). No relationships noted for any abuse and BMI among males. CSA increased odds of major depressive disorder (MDD) (B = 1.10, 95%CI: 0.66–1.54) and generalized anxiety disorder (GAD) (B = 0.29, 95%CI:0.59, 1.98). MDD increased odds of BMI at wave 4 (B = 1.35, 95%CI: 0.52–2.18) but not wave 5 (B = -0.09, 95%CI -0.5,0.32). No mediation by GAD was found. For males, no associations between MDD or GAD and BMI were found. Evidence found for an indirect relationship between sexual abuse and BMI (wave 4) via MDD. Relationship between MDD and BMI (wave 4) was moderated by sexual abuse only for females.	Socio-economic status of parents, ethnicity of child, BMI at previous waves, age.	Major depressive disorder (MDD) Generalized anxiety disorder (GAD)	(1) CSA(For the relationship between MDD and BMI) (2) Sex/Gender	CSA among females was associated with elevated BMI at the end of adolescence and a higher increase in BMI during young adulthood. This relationship was mediated by MDD at the end of adolescence. For females, CSA also moderated the relationship between MDD and BMI at the end of adolescence. Overall, the relationship between MDD and BMI at the end of adolescence was particularly present among sexually abused females.
Li (2021)	Higher initial cortisol level in childhood and attenuated cortisol growth rate (HPA axis attenuation) were associated with an accelerated rate of BMI accumulation across development. Mediation analysis: (1) Initial cortisol level (*β* = 0.09, *p* = .02) and cortisol growth rate (*β* = - 1.61, *p* = 0.005) in separate models had a statistically significant BMI linear slope effect. Inclusion of cortisol growth rate yielded an insignificant (CSA x BMI) linear slope coefficient. (2) Indirect effect of CSA on adult obesity status via cortisol growth rate was statistically significant: (*β =* 0.38, 95% CI: 0.02–1.06). Direct path of CSA to adulthood obesity status, controlling for cortisol growth rate: (*β =* 1.07, p = 0.03).	Steroid medication (contraceptives), race and ethnicity, parity, depression, disordered eating history.	Serum cortisol level	No	Findings indicate the relationship between HPA axis dysregulation (cortisol attenuation) and increased risk of later obesity and elevated BMI among CSA survivors who are female, compared with non-abused females.
Martin Martin (2010)	1) 21% of 29 girls < 5 had low ’emaciated’ weight for height in those who suffered physical abuse in those < 5; while none among those 26 who had suffered sexual abuse. chi-square 6.08, p<0.01 2) 48% of 29 girls< 5 had low height for age in those who suffered physical abuse in those < 5; while 3 (12%) among those 26 who had suffered sexual abuse. chi square 8.66 p<0.005	No	No	No	Girls <5 years who were victims of physical abuse were more likely to be underweight and stunted than those who were victims of sexual abuse (no adjustment), chi square only. Among children ages 5–11, girl victims of physical abuse were more likely to be underweight (by BMI), stunted, than girls victims of sexual abuse, while girls victims of sexual abuse were more likely to be overweight or obese (than those who had suffered physical abuse).
Noll (2007)	OR estimates for childhood/early adolescence (6–14): (1) Comparing abused adolescents to non-abused adolescents: 1.25 (-0.05, 3.00), p = 0.52 Middle to late adolescence (15–19): (2) Comparing abused adolescents to non-abused adolescents: 2.03 (0.54, 4.60), p = 0.09 Young adulthood (20–27): (3) Comparing abused adolescents to non-abused adolescents: 2.85 (1.06, 4.64), p = 0.009 (4) Abused female participants acquired BMI at a steeper rate on average than non-abused counterparts and abused female respondents were heavier throughout development, after control.	Adjusted for minority status (nonwhite) and parity	No	(1) 3 distinct age periods: childhood and early adolescence (6 to 14 years), mid to late adolescence (15 to 19 years); and young adulthood (20 to 27 years)	By young adulthood, sexually abused females were more likely to be obese compared to the comparator group and abused females on average acquired body mass at a steeper rate from childhood to young adulthood, compared to their non abused comparators
Peckins (2019)	Beta estimates Boys: Sexual abuse and BMI trajectory: 2.31 (-11.76, 16.38) Cortisol reactivity*Sexual abuse: 8.63 (-23.41, 40.67) Girls: Sexual abuse and BMI trajectory: -2.67(-15.22,8.89) Cortisol reactivity*Sexual abuse: -9.98(-40.51,20.55) Significant interaction between type of maltreatment*cortisol reactivity* quadratic change in percentile of BMI over time	Race/ethnicity, pubertal stage, psychological functioning (depression, anxiety)	No	(1) Moderation by cortisol reactivity at Time 1	Among girls, cortisol reactivity moderated the relationship between type of maltreatment and quadratic change in BMI: a steeper quadratic increase in BMI was observed among sexually abused girls with low levels of cortisol. At high levels of cortisol, sexually abused girls did not differ from the comparator. No associations noted for males.
Schiff (2021)	Odds ratio Estimates: Sexual abuse on BMI using fixed effects regression was not significant for females. (OR: 0.22, 95%CI: 0.02, 2.76).	Number of children living in household and family poverty	No	(1) Sex/Gender	Overall, youth at higher ACE cumulative scores faced greater odds of obesity, compared with youth at lower ACE cumulative scores. However, gender stratified associations were not significant for males or females. The association with CSA did not reach statistical significance for females.
Schneiderman (2012)	Interaction effect odds ratio and 95% confidence intervals from logistic regression: (1) Sexual Abuse x gender (male = ref) and overweight/obese: 0.32 (0.09, 1.19) p = 0.09 (2) Sexual Abuse x gender (male = ref) and obese: 0.24 (0.06, 0.996), p = 0.05*	Race/ethnicity pubertal stage, psychological functioning (depression, anxiety)	No	(1) Sex/Gender	Significant interaction between sexual abuse and gender, meaning that being sexually abused reduced the odds being obese among females, compared to maltreated adolescents who were unexposed to sexual abuse
Schneiderman (2014)	For Females: Linear Slope Beta Estimate: (1) Sexual abuse vs non-abused comparator: -2.16(1.66) Quadratic Slope Beta Estimate: (2) Sexual abuse vs non-abused comparator: 0.29(0.14)* Girls exposed to sexual abuse has a slower increase in BMI percentile (compared to those unexposed), until 16–17 years of age, when BMI percentile was higher than girls in the comparison group	Race/ethnicity pubertal stage, psychological functioning (depression, anxiety)	No	(1) Sex/Gender	Girls exposed to a history of maltreatment had different BMI grown trajectories than comparator/unexposed girls. At 15–16 years old, comparator group BMI decreased and girls with sexual abuse had higher BMI (analyzed on a quadratic scale).
Soares (2018)	Supplementary Gender Stratified Analysis (mean difference in outcome, exposed vs unexposed), selected results: Among Female Adolescent in UK Cohort: Sexual abuse and BMI at 15: -1.16 (-2.86, 0.54) Sexual abuse and waist circumference at 15: -1.87 (-6.44, 2.70) Sexual abuse and BMI at 18: -1.14 (-3.12, 0.83) Sexual abuse and fat mass index at 18: -0.54 (-2.25, 1.18) Among Female Adolescent in Brazil Cohort: Sexual abuse and BMI at 15:-0.82 (-1.91, 0.28) Sexual abuse and waist circumference at 15: -1.80 (-4.14, 0.54) Sexual abuse and BMI at 18: -0.63 (-1.98, 0.73) Sexual abuse and fat mass index at 18: -0.55 (-1.88, 0.77) Sexual abuse and android fat at 18: 0.11 (-0.30, 0.53) Domestic violence and BMI at 15: -0.02 (-0.51, 0.47) Domestic violence and waist circumference at 15: 0.05 (-1.00, 1.10)	All models adjusted for family income, maternal education, maternal age, maternal smoking during pregnant, maternal pre pregnancy BMI, birth weight skin color	No	(1) Sex/Gender	Few significant associations were noted between adverse child experiences and adiposity during adolescence.
Soares (2021)	Sex differences noted for association between sexual abuse and BMI at 18 years (p-value for interaction = 0.052). Significant associations noted only in men (BMI, β 2.15 kg/m2; 95% CI, 0.62–3.68) but not women (BMI, 0.33 kg/m2; 95% CI, −0.26 to 0.93). For females, no significant association between sexual abuse and cholesterol (mmol/L): B = 0.00, 95% CI (-0.13, 0.13), HDL (mmol/L): B = 0.02, 95%CI: (-0.04, 0.07), LDL (mmol/L): B = 0.01 (-0.11, 0.12). For females, significant associations between sexual abuse and triglycerides (mmol/L): B = 0.96, 95%CI: 0.90, 1.03 and glucose B = 1.00, 95%CI (0.98, 1.01).	Age, sex, ethnicity, maternal education, paternal education, and parental social class	No	(1) Sex/Gender	Higher BMI at age 18 was associated with sexual abuse in the unstratified sample. However, this was only true among the male portion of the stratified sample, and was not evident in the female stratified sample.
Pathway 1b: Exposure to intimate partner/dating violence and/or sexual violence during adolescence and later elevated BMI					
**Study**	**Adjusted Result(s)**	**Potential Confounders**	**Mediators**	**Moderators**	**Summary of findings/results**
Anderson (2015)	Bivariate analyses conducted for forced sex and overweight: History of sexual assault and overweight among females: 1.18 (0.99–1.41)	Not adjusted	No	(1) Sex/Gender: (2) Interaction of forced sex history and overweight on suicidal risk	Results of the univariate analysis did not indicate a statistically significant relationship between sexual assault and overweight during adolescence
Clark (2014)	Betas for Independent effects OLS regression among females: (1) Dating violence and BMI: 0.4 (0.1 to 0.7); CSA by parents/caregivers: 0.6 (-0.1 to 1.3) Betas for Interaction effect of sexual abuse and dating violence on BMI among females: (1) Exposed to dating violence and sexual abuse: 1.3 (0.3 to 2.3); exposed to dating violence but not sexual abuse: 0.3(0.0 to 0.6)	(1) Adjusted for baseline age. race/ethnicity, parental education, family history of obesity, BMI, and depressive symptoms (2) Adjusted for sexual abuse child maltreatment and baseline age. race/ethnicity, parental education, family history of obesity, BMI, and depressive symptoms	No	(1) Frequency of abuse and neglect perpetrated by parents/adult caregivers, occurring before 6th grade (maltreatment, inadequate supervision, physical neglect, sexual abuse)	Dating violence was a significant predictor of increased BMI over time among females and exposure to childhood abuse magnified the BMI increase among those exposed to dating violence. No associations in males were noted.
Souza Marques (2021)	Odds ratio estimates among girls: (1) Family physical violence and Overweight/Obesity: 0.97 (0.76, 1.24) (2) Rape and Overweight/Obesity: 1.64 (1.15, 2.33)	For (1) and (2): age, skin color/race, mother’s educational level, living with the mother, type of school, school shift	No	(1) Sex/Gender	Rape was associated with nutritional status in adolescents of both sexes; physical family violence was not
Souza Marques (2022)	Odds ratio estimates among girls: Sexual violence exposure on outcome of overweight/obesity versus normal BMI: 1.64 (1.15–2.33), p = 0.006 In males, sexual violence exposure was associated with increased odds of underweight compared to normal weight. No associations with family physical violence and BMI.	Age, skin color/race, mother’s educational level, living with the mother, type of school, school shift	No	(1) Sex/Gender	Rape (not family physical violence) was associated with excess body weight among females (older than 13 years) and being underweight among males (older than 13 years).
Veldwijk (2012)	Odds ratio estimates among girls: (1) Underweight (vs normal weight) and sexual abuse: 0.83(0.71, 0.97) (3) Overweight (vs normal weight) and sexual abuse: 0.95(0.77, 1.16) (4) Obesity (vs normal weight) and sexual abuse: 1.11(0.66, 1.87)	Ethnicity, educational level, parental communication, and the remaining subtypes of abuse	No	(1) Sex/Gender	Underweight (compared to normal weight) was associated with less odds of sexual abuse
Pathway 2: Exposure to child marriage and undernutrition					
**Study**	**Adjusted Result(s)**	**Potential Confounders**	**Mediators**	**Moderators**	**Summary of findings/results**
Tiruneh (2021)	Odds ratio: Ever married (during adolescence) and anemia: 1.54 (1.07, 2.22) Early childbearing and anemia: 1.22 (0.77, 1.95)	Religion, urban/rural residence, wealth index, education, employment, currently pregnant, currently breastfeeding, current use of contraception, BMI, health facility uses in past year.	No	No	There was a significant association between early marriage and anemia, measured at the individual level.
Wells (2022)	Interaction for pregnancy and marriage age in the BMI model (p = 0.027) Interaction for pregnancy and marriage in the MUAC model (p = 0.06) Multi-variate linear regression models for BMI and mid upper arm circumference (MUAC) Outcomes at Endpoint: Younger age at marriage (1) increases odds of BMI at endpoint and MUAC at endpoint and (2) decreases odds of chronic energy deficiency at endpoint. Ex) Married ≤ 14 (compared to >17): *β* = 0.33, 95%CI: 0.08, 0.58, p = 0.009 Younger age at first pregnancy (1) reduces the odds of BMI and MUAC during early pregnancy and (2) increases odds of chronic energy malnutrition at endpoint. Ex) Pregnancy at ≤ 15 (compared to >19): *β* = -1.08, 95%CI: -1.08, -0.78, p<0.001	Caste, maternal and paternal education, land ownership, asset quartile, study arm and cluster In addition, marriage age model controlled for pregnancy age and age at pregnancy model controlled for marriage.	No	(1) Age at first pregnancy	The association between age of marriage and mother’s nutritional status varied depending on pregnancy age. For women who first became pregnant at age 15 or less, marriage at earlier ages resulted in significantly lower BMI at endpoint compared to those who were married later. For women who had their first pregnancy at age 19 or older, earlier marriage was associated with higher BMI.
Pathway 3: Exposure to childhood abuse (including sexual) and reduced height and leg length					
**Study**	**Adjusted Result(s)**	**Potential Confounders**	**Mediators**	**Moderators**	**Summary of findings/results**
Denholm (2013)	OLS Beta estimates and SE for Child Maltreatment and standard deviation score of height and leg length at 16 years: Number of types of abuse (0–3): 0.04(0.03) Witnessed violence (0–1): 0.07(0.05) Maltreatment score (0–7): 0.02(0.01) Among females, sexual abuse and adjusted leg length at 16: 0.08(0.09) Sexual abuse and height at 7years: -0.09(0.10) Sexual abuse and adult height: 0.12(0.09)	Adjusted for parental height, birthweight, maternal smoking, social class, infant feeding, household crowding, tenure and amenities, and disability.	No	(1) Sex/Gender: (2) Age at follow up (7, 11, 16, Adult)	No significant association between abuse and witnessing abuse among females and height and leg height by 16 years. However, overall, the study found that childhood neglect and household dysfunction assessed at 7 years was associated with shorter stature among both males and females

### Pathway 1: Exposure to sexual abuse during childhood and adolescence and later elevated BMI (i.e., overweight, obesity) or increased adiposity

#### Pathway 1a: Exposure to sexual abuse by trusted adults during childhood and adolescence and later elevated BMI (i.e., overweight, obesity) or increased adiposity

Overall, the evidence suggests that CSA perpetrated by parental/caregiver figures is associated with later increases in BMI, including overweight/obesity in late adolescence and early adulthood. Ten studies specifically explored this pathway, five of which were conducted in children referred for maltreatment in the U.S. [56–59], with three reporting on the same longitudinal study cohort of maltreated children in Los Angeles [L.A.] [57–59]. In this longitudinal cohort study, there was no increase in BMI associated with exposure to sexual abuse during childhood and early adolescence [58], but rather increased BMI trajectory was apparent only during middle to late adolescence when compared with girls exposed to no violence or physical violence only [59]. Another U.S. based study of children referred for maltreatment similarly found that by young adulthood, females who had experienced sexual abuse in childhood were more likely to be obese, compared with those not abused [56]. Further, Peckins et al.’s (2019) [57] analysis on the L.A. cohort suggests that response to stress measured through cortisol reactivity may act as an intermediary between exposure to CSA and later increases in BMI trajectories (in girls but not boys). Using longitudinal data, Li et al., (2021) tested whether HPA axis attenuation (high values of initial basal cortisol levels and diminished cortisol increases) accounts for the elevated risk of obesity among girls who experienced CSA, compared to unexposed girls [41]. When holding history of depression, disordered eating, and use of oral contraception (among other commonly controlled for covariates) constant, results indicated that attenuated cortisol growth rate mediated effect of CSA on elevated BMI accumulation and elevated adult obesity. Further considering mediators, Elsenburg et al., (2022) tested whether major depressive disorder (MDD) and generalized anxiety disorder (GDD) mediated the relationship between CSA and BMI among male and female young adults [71]. Among females, CSA was positively related to BMI at wave 5 only and CSA was positively related to MDD and GAD, which in turn were positively related to BMI at wave 4. Among males, there was no relationship between CSA and BMI or the mental health mediators and BMI. Together these studies suggest that the effects of sexual abuse on elevated BMI emerge during late adolescence and young adulthood for females. Further, low cortisol reactivity could be a risk profile for greater BMI in late adolescence among girls exposed to child sexual abuse, compared to those unexposed and those exposed to physical abuse. When longitudinal BMI data was analyzed cross sectionally, insignificant associations between CSA and BMI were noted [67, 69, 70] and when cross sectional data were used relationships indicating CSA has a protective effect on elevated BMI [58].

#### Pathway 1b: Exposure to intimate partner/dating violence and sexual violence during adolescence and later elevated BMI

There is some evidence highlighting the risk of elevated BMI following exposure to sexual violence (perpetrated by any perpetrator, not specific to trusted adults such as parents/caregivers) and intimate partner/dating violence. In total, five studies examined the pathway between exposure to intimate partner/dating violence and sexual assault/rape perpetrated by intimate and non-intimate partners and elevated BMI among girls. The strongest evidence emerged from Clark et al., (2014) [60] who explored the pathway between exposure to dating violence/IPV (physical and sexual combined) and elevated BMI, finding that exposure to intimate partner/dating violence was positively associated with increasing BMI. Exposure to CSA and adolescent exposure to intimate partner/dating violence was further positively associated with elevated BMI. Marques et al. (2021) examined the relationship between exposure to rape and overweight/obesity among female adolescents over 13 years in Brazil, concluding that rape was positively and significantly associated with overweight and obesity among girls [66]. Veldwijk et al., (2012) [63] analyzed the relationship between sexual assault perpetrated by adults or peers and the presence of underweight, overweight, and obesity among adolescents between 13 and 16 years in the Netherlands. The only significant association found indicated that exposure to sexual abuse lowered the odds of being underweight. Using cross-sectional data from Brazil, Souza Marques et al., (2022) reported that for adolescent girls (attending school), forced sexual intercourse was positively related to overweight/obesity versus normal BMI and for adolescent males, forced sexual intercourse increased the odds of underweight versus normal BMI [66]. The only null result came from Anderson et al.’s (2015) [75] study; these results stemmed from a simple bivariate analysis between forced sex and overweight.

### Pathway 2: Exposure to child marriage and undernutrition

Only two studies examined child marriage and measures of undernutrition (anemia, MUAC, BMI, chronic energy malnutrition [BMI < 18.5kg/m^2^]) [61, 68]. We found one study reporting an association between girl child marriage and anemia. This study analyzed the Ethiopian national Demographic and Health Survey data, finding that a history of child marriage among girls was significantly and positively associated with anemia (low hemoglobin) in adolescence [68]. The study did not examine history of childbirth. A second study using data from Nepal, investigated the relationship between age at marriage and age at first pregnancy and the outcomes of BMI, MUAC, and chronic energy deficiency [61]. The interaction between age at marriage and age at first pregnancy was significant in the BMI outcome model and marginally significant in the MUAC outcome model. For women who first became pregnant at age 15 or less, marriage at earlier ages resulted in significantly lower BMI at endpoint, compared to those who were married later. For women whose first pregnancy occurred at age 19 or higher, earlier marriage was associated with higher BMI. Pregnancy at age 15 or lower was positively associated with chronic energy deficiency, compared to pregnancy at 19 years or older.

### Pathway 3: Exposure to childhood abuse (including sexual) and reduced height and leg length

One study investigated the relationship between child abuse (including sexual abuse) perpetrated by parents/caregivers and reduced height and leg length among girls [62]. The study found that leg length at age 16 was not associated with number of abuse types experienced, witnessing violence, or with overall maltreatment scores among females. Further, sexual abuse was not significantly associated with adult height.

## Discussion

We conducted a REA to synthesize quantitative evidence describing associations between girls’ exposure to GBV and indicators of nutrition status during childhood and adolescence. Overall, the evidence base is limited: only 18 studies met our inclusion criteria and most studies examined either CSA or IPV/dating violence with later overweight/obesity/measures of adiposity. Our review indicates the lack of studies investigating the potential impacts of exposure to GBV on indicators of childhood and adolescence nutrition status among females; in particular, nutrition outcomes beyond those calculable with measures total body height and weight, indicators of key nutrient levels (including iron deficiency), or more sensitive indicators of growth during specific developmental periods. Beyond CSA, dating violence/IPV, and child marriage, no other forms of GBV were studied in relation to girls’ nutrition indicators. Another form of GBV that warrants empirical attention is the preferential feeding of boys which could be relevant in humanitarian/fragile settings where there may be high levels of food insecurity [46, 76–78]. Expansion of this evidence base in LMIC is also needed, with appropriate study designs and analytical procedures that account for high baseline levels of malnutrition and multi-level risk factors for malnutrition.

Our synthesis elucidated three potential pathways linking exposure to GBV and indicators of subsequent childhood and adolescence nutrition status among females:

1. Exposure to sexual abuse by a) parents/caregivers during childhood (CSA) and later elevated BMI (i.e., overweight, obesity) or increased adiposity, b) IPV/sexual violence during adolescence and later elevated BMI (i.e., overweight, obesity) or increased adiposity. The evidence base most clearly and consistently supported this pathway, given most studies analyzed sexual violence and BMI/obesity/overweight/increased adiposity outcomes.
2. Exposure to child marriage and undernutrition (amenia, chronic energy deficiency, and MUAC). There is emerging evidence supporting this pathway. However, additional research is needed to replicate the findings of the two studies investigating child marriage and undernutrition. Importantly, age at first pregnancy appears to be a consequence of girl child marriage or an impetus for girl child marriage that has negative nutritional consequences for adolescent females.
3. Exposure to childhood abuse (including sexual) and reduced height and leg length. The evidence base is inconclusive regarding this pathway, given only one study with insignificant results was found.

Among the included studies, the strongest and most consistent evidence supports the relationship between elevated BMI in late adolescence and early adulthood and exposure to sexual abuse during childhood (CSA) or adolescence (intimate partner violence/sexual assault/rape). Childhood and adolescent exposure to sexual violence, perpetrated by parents/caregivers and intimate partners, is an important predictor of nutrition status indicators in childhood and adolescence among females. Understanding the biological mechanisms that underlie this association may help guide further research regarding the consideration of intermediary variables, timing of causal effects, prevention efforts, and actionable periods of intervention.

CSA and exposure to IPV in adolescence are examples of ACEs, however health impacts are largely measured in adulthood and nutrition outcomes are less considered compared to chronic disease, mental health, and substance use. This is an important research gap given that findings from this REA indicate nutrition outcomes such as adolescent obesity are associated CSA, IPV, and sexual assault/rape exposure during childhood and adolescence. To further understand mechanisms that link GBV exposure in childhood/adolescence and the early emergence of nutrition disorders, additional research that considers childhood and adolescence nutrition outcomes is needed.

Our findings highlight that sexual violence, either perpetrated by parents/caregivers during early or middle childhood or by intimate partners or strangers later on in adolescence, is a stress-inducing form of trauma which engenders nutrition-related consequences in childhood and adolescence (as seen in this review), and into adulthood [15]. Previous studies have linked ACEs, including CSA and sexual violence, to stress response dysregulation [41, 79–81]. Like other ACEs, the biological mechanism linking sexual violence to elevated BMI likely involves the hypothalamic-pituitary-adrenocortical (HPA) axis (although, other pathways may also be involved). The HPA axis modulates the stress response by regulating the secretion of cortisol in response to environmental conditions perceived as being stressful and beyond an individual’s capacity to cope [7, 82, 83]. Exposure to sexual violence in early childhood and adolescence can serve to activate the HPA-axis in a manner that can negatively affect health outcomes related to nutrition, mental health, and immunity [79]. Long term or chronic activation of the HPA-axis from exposure to GBV and other adversities results in an attenuated diurnal decrease in cortisol or dampened/attenuated cortisol reactivity [57, 82], thus chronic exposures to GBV may be especially harmful.

Higher values of initial cortisol levels followed by a lowered rate of cortisol increase is characteristic of HPA axis dysregulation. Dampened or attenuated cortisol reactivity occurs when the HPA axis adapts to prolonged stress-induced cortisol secretion. The downregulation of basal cortisol secretion is a protective mechanism to mitigate the negative consequences of elevated cortisol. For adolescent girls, cortisol dampening/attenuation can negatively interact with normal pubertal physiological maturation, increasing the risk of developing overweight/obesity and adiposity. Biochemical pathways (metabolic disturbances, altered insulin signaling, inflammation induced damage to leptin and insulin receptors), behavioral pathways (increased impulsivity and related overeating), and/or the interaction between biochemical and behavioral pathways may explain how cortisol attenuation/dampening is related to overweight/obesity and adiposity [41, 84–86]. We did not locate any studies that considered eating patterns as intermediary variables between GBV and elevated BMI/overweight/obesity/adiposity, limiting a nuanced understanding of the behavioral pathway and its connection to the biochemical pathway.

Research focusing on childhood GBV has demonstrated that among girls who have experienced CSA, while initial cortisol levels following sexual abuse exposure may be high, there is a developmental transition to lower levels of cortisol in early adulthood via cortisol attenuation [87]. More recently, research supports associations between cortisol attenuation and CSA exposure in later childhood and early to mid-adolescence (ages 6–16) among females, wherein serum basal cortisol levels further decreased longitudinally into adulthood [41]. Cortisol attenuation over time (from childhood to adulthood) was also associated with an accelerated rate of BMI accumulation across development (late childhood, adolescence, and early adulthood) and completely mediated the effect of CSA on accelerated BMI accumulation over development [41]. In the articles we reviewed, exposure to sexual abuse in school aged girls was associated with elevated BMI in late childhood, peri-puberty (ages 6–11), and late adolescence into early adulthood. Importantly, we noted that in Martin and Martin, (2010) [64], Peckins et al., (2019) [57], Elsenburg et al., (2022) [71], and Souza Marques et al., (2022) [66], such relationships were only noted among girls, thereby suggesting the importance of conducting a sex-stratified analysis. Other studies have also examined the nutritional impact of adverse childhood life events, including one systematic review focused on the relationship between ACEs more generally and childhood obesity among both boys and girls, which highlighted that sexual abuse appears to have a greater effect on childhood obesity than other ACEs and particularly among girls [43].

The currently synthesized evidence base and the wider literature presents mixed results for sex and/or gender differences in HPA axis attenuation following exposure to chronic stressors and nutrition outcomes involving elevated BMI/obesity/overweight/adiposity. Additionally, there is paucity of tested biological and social mechanisms that account for any sex/gender differences noted. Compared to males, females may be more susceptible to stress-related disorders that impact eating behaviors [88]. Adolescent females also have estrogen-related elevated central adiposity due to hormonally regulated differences in fat distribution, with females developing higher subcutaneous and gluteofemoral fat [89]. In addition, GBV exposure distribution may differ by gender: girls are at greater risk for poly-victimization (cumulative exposure to CSA, sexual IPV/dating violence, and non-partner rape across childhood and adolescence) compared to boys, thereby translating to a dysfunctional stress responses characterized by cortisol attenuation and internalized mental health sequela [90, 91].

Future research investigating the relationship between exposure to CSA and IPV or dating violence among females could consider the inclusion of theoretically relevant intermediary variables. Other potential mechanisms beyond dampened/attenuated cortisol reactivity may also include mental health mediators, given that CSA can lead to mental health disorders and negative affect. For example, ‘emotional overeating’ at age 10 has been associated with exposure to adverse events in younger children [92]. Gaining weight by overeating may also be perceived as a means of deterring unwanted sexual attention/advances among girls previously exposed to CSA [41, 93]. It is important to also consider the role of eating disorders, PTSD and depression as mediator or effect modification variables. Only Elsenburg et al., (2022) considered the mediating role of major depressive disorder and generalized anxiety disorder on the relationship between CSA and BMI among young adults, reporting that CSA is positively associated with major depressive disorder which is in turn positively related to BMI for females but not males [71]. Other research has shown that among survivors of sexual violence, a transition from acute cortisol hyper-reactivity to prolonged hypo-reactivity is characteristic of post-traumatic stress disorder among females (PTSD) [79]. Depression is also of relevance: in a meta-analysis, Danese and Tan (2014) established that when estimates were adjusted for current depression, the relationship between childhood maltreatment (including sexual abuse) and obesity became insignificant [9]. Thus, studies can consider depression as a potential intermediary variable given it is likely a consequence of GBV and can affect nutrition outcomes. Lastly, research could also consider protective factors among children exposed to ACEs, including GBV, and how protective factors such as, quality education, presence of role models, and school/extracurricular engagement, and mental health supports [94, 95], can moderate the development of adverse nutrition outcomes.

We also noted that the effect of childhood exposures to sexual violence on elevated BMI, overweight/obesity/adiposity manifests during sensitive periods of adolescent development into early adulthood. Scholarship has focused mainly on elucidating at what developmental periods childhood maltreatment generally impacts elevated BMI and obesity, with late adolescence/early adulthood emerging as a sensitive period [9, 96]. For example, Sokol et al., (2018) [97] tested childhood exposures to physical, sexual, and emotional abuse and found that these yielded different BMI growth trajectories between the ages of 13 to 28 years for males and females. Importantly neither of these studies included examination of pre-pubertal growth trajectories, thus potential earlier impacts are unknown. In particular, girls’ exposure to sexual abuse between 13 and 17 years was positively associated with BMI in late adolescence to early adulthood, from 19 to 24.5 years of age [96]. A meta-analysis of over 40 studies reported a higher risk of obesity in adulthood, following exposure to child maltreatment, not childhood/adolescence [9]. However, the meta-analysis included few studies that examined exposure to sexual abuse with nutritional indicators measured during childhood and adolescence, as the majority of studies examined nutritional status measured in adulthood [9]. Thus, further research examining exposure to sexual violence with nutrition indicators measured during earlier developmental periods (including prior to and through adolescence into early adulthood) is needed.

Overall, we recommend further clarifying the linkage between GBV and nutrition during childhood and adolescence in females. There is a research-related gap in theorizing and empirically testing mechanisms that aim to explain how and why GBV against girls negatively impacts their nutritional status during later childhood and adolescence. The present REA found evidence to support associations between exposure to sexual violence during childhood and adolescence and elevated BMI/overweight and obesity later in childhood and adolescence. However, nuanced understandings of the causal mechanism underlying this pathway are limited. Given that adolescence represents a period of potential growth catch up and can coincide with pregnancy and child rearing [98], it is important that future studies consider measuring nutritional status among adolescent girls.

From a methodological standpoint, future research should specifically examine exposure to sexual violence when examining nutrition indicators in younger children. Sexual violence exposure can be disaggregated from other ACEs and measured by timing of exposure [infancy, early childhood, middle childhood, or adolescence; or by pubertal stage] and perpetrator type [family member/relative/caregiver, intimate partner, peer, stranger]). Important mediator variables include measures of behavioral changes (internalizing [emotional eating, low self esteem] and externalizing [aggression]), mental health symptoms and disorders (depression, PTSD, eating disorders), impulsivity, and dampened/attenuated cortisol reactivity. Further research can elucidate sensitive periods for anthropometric measures vis-à-vis exposure to sexual violence. Prospective longitudinal collection of informative biomarkers beginning early in childhood among potential at-risk populations will be key for improving our understanding of the relationship between exposure to sexual violence, a dysregulated stress response (including measures of behavior and mental health), and later elevated BMI. Research aiming to investigate the effect of exposure to sexual violence during childhood and adolescence on elevated BMI should consider longitudinal study designs that measure BMI into early adulthood to include sensitive periods of development. Sex-stratification of data could also elucidate sex and gender specific mechanisms, particularly given the sex-based differences in timing of developmental stages. Greater attention should be given to the temporal sequence of variables. For example, measuring mental health symptoms/disorders in parallel with nutrition outcomes can obscure the correct temporal sequence between mental health intermediaries and nutrition outcomes. Accurately capturing cortisol dampening/attenuation requires that investigators measure diurnal serum cortisol variation according to the circadian rhythm.

Our findings should be interpreted in light of some limitations. We conducted single screening of titles and abstracts (a single reviewer determined whether to include or exclude). Although in line with guidance on how to adapt systematic review methodology for an REA [99], single screening of titles and abstracts may have excluded relevant studies. Further, disparate use of outcome measures may mean that results from specific studies are not directly comparable. Lastly, we excluded articles in languages other than English and Spanish due to resources and time-constraints.

## Conclusion

This REA identified a small evidence base of 18 quantitative studies investigating the association between GBV perpetrated against girls and subsequent child and adolescent nutrition outcomes. Only two studies investigated the relationship between child marriage and undernutrition, indicating the need for greater attention paid to the nutritional consequences of child marriage on girl brides and the importance of considering age at first pregnancy within the context of girl child marriage. Most included studies investigated the relationship between the outcomes of overweight/obesity/increased adiposity and the exposures of CSA, IPV, and dating violence. We recommend consideration of relevant intermediary variables and timing of effects, pertaining to the biologic mechanisms underlying the relationship between childhood GBV exposure and overweight/obesity. Future research is needed to not only better understand the mechanisms that underlie the relationship between childhood sexual violence exposure and overweight/obesity, but also consider other GBV exposures such as child marriage, the preferential feeding of boys on nutrition outcomes beyond overweight/obesity, and additional research in LMIC, resource poor and humanitarian settings.

## Supporting information

S1 Appendix Appendix A: Search domains and terms for all databases. (DOCX)

S2 Appendix Appendix B: Rapid evidence assessment protocol. (DOCX)

S1 Checklist Preferred Reporting Items for Systematic reviews and Meta-Analyses extension for Scoping Reviews (PRISMA-ScR) checklist. (DOCX)
