# Supplementary material for: Childhood and adolescent nutrition outcomes among girls exposed to gender-based violence: A rapid evidence assessment of quantitative research
Source: PLoS One. 2023 Feb 16;18(2):e0281961. doi: 10.1371/journal.pone.0281961 (PMC9934406; doi:10.1371/journal.pone.0281961)
Supplement: S2 Appendix — (DOCX) [file pone.0281961.s002.docx]

**Appendix B: Rapid Evidence Assessment Protocol**

**Background:**

This rapid evidence assessment (REA) will focus on identifying, describing, and appraising pathways between gender-based violence and childhood nutrition outcomes. In doing so, this REA responds to efforts being made to incorporate GBV risk mitigation activities within the nutrition sector of humanitarian response. Both direct pathways involving childhood and adolescent exposures to GBV and indirect pathways involving GBV experienced by parents/caregivers will be included.

To identify relevant pathways, we will apply systematic review methodology in a manner that is adapted to the approach and needs of a rapid evidence assessment. This entails combining the structured and rigorous search and assessment components of a systematic review with adaptations and adjustments at most stages of the process to promote completion of the review, in a way that is focused on applicability to policy and programmatic needs, in a short timeframe. The methodology will occur in the following stages: (i) Database search to locate relevant sources, (ii) screening of titles and abstracts, (iii) full text review, (iv) data extraction/charting, (v) quality assessment.

Methods of adapting systematic review methodology in our proposed REA are: splitting review of title/ abstract and full text among the team members during screening, extraction, and quality assessment and restricting our searches to a limited number of databases. The inclusion and exclusion criteria will also be determined iteratively, depending on the volume of sources and UNICEF priorities. In doing so, we are maximizing the benefits of quick evidence generation as relevant to UNICEF priorities and stakeholders while also maintaining a rigorous methodological approach.

1. **Review questions**

**Aim:** To review and synthesize the quantitative evidence describing the associations between gender-based violence against girls and girls’ nutrition outcomes, with a particular focus on humanitarian contexts.

**Aim:** To review and synthesize the quantitative evidence describing the associations between intimate partner violence against a maternal caregiver and children’s nutrition outcomes, with a particular focus on humanitarian contexts.

**Relevant definitions:**

**Gender-based violence against girls:**

- 1. Child marriage: We define child marriage as any formal or informal union in which the girl is under 18 years of age
  2. Early marriage: We define early marriage in the same way as child marriage; the two terms are sometimes used interchangeably, therefore we will include the term early marriage in our search terms
  3. Forced marriage: We define forced marriage as a situation in which “one or both parties have not personally expressed their full and free consent to the union” [UN OHCHR]. We consider child marriage to be a form of forced marriage, and will include it in the search terms and include studies focusing on forced marriage of girls under the age of 18.
  4. Preferential feeding: We define preferential feeding as food and nutrition-related caring practices that advantage boys over girls.
  5. Sexual violence: We define sexual violence as “being forced, coerced or threatened to perform any unwanted sexual act; this could include rape, attempted rape, unwanted sexual touching or non-contact forms of sexual violence” (WHO 2021). We include this separately from IPV as girls may experience sexual violence from a range of perpetrators, and we include here sexual violence perpetrated by any family member apart from a partner/ husband, teachers, persons in other authority roles, strangers, etc.
  6. Intimate partner violence: We define intimate partner violence [IPV] as “any behaviour by a current or former male intimate partner within the context of marriage, cohabitation or any other formal or informal union, that causes physical, sexual or psychological harm” (WHO 2021). We will include the following types of IPV: physical, sexual, psychological (including search terms for verbal and emotional), controlling behaviors.
  7. Girls: We define girls as any female under the age of 18. We will include the term adolescent in the search strategy to ensure that studies that focus on female adolescents are included.

**Intimate partner violence against a maternal caregiver:**

The second objective in the REA focuses specifically on IPV as the form of indirect GBV to which children (boys and girls under the age of 18) may be exposed. Non-partner violence, including non-partner sexual violence, is of significant concern in humanitarian contexts. However, the global epidemiology and evidence-base concerning the associations between maternal violence exposure and child outcomes indicates that a focus on IPV is warranted. Evidence indicates exacerbation of IPV in humanitarian contexts, and specific elements of the epidemiology of IPV (including repeated and overlapping exposures, patterns of severity, etc) indicate that there are plausible and consistent associations between IPV and physiological, psychosocial and behavioral pathways that impact children’s nutrition outcomes.

In the second objective of the REA, we define a child as any boy or girl under the age of 18.

1. In-utero exposure: We define in-utero exposure to IPV as any case where a fetus is exposed to IPV through the female caregiver’s exposure to IPV while pregnant.
2. Household exposure: We define household exposure to IPV as any context where a female caregiver of a child is exposed to IPV, including but not limited to if a child witnesses IPV in the household. In the case of household exposure, associations between household IPV exposure and children’s nutrition outcomes may be due to female caregivers’ constrained choices and practices in an environment affected by IPV, or due to child-level psychological and physiological changes due to direct witnessing of IPV.

**Humanitarian contexts:**

The REA will include evidence generated in, but not limited to, humanitarian contexts.

1. Humanitarian contexts: We take the following definition of humanitarian contexts from UNICEF’s Core Commitments for Children (<https://www.corecommitments.unicef.org/ccc-1-1>): “A humanitarian crisis is defined as any circumstance where humanitarian needs are sufficiently large and complex to require significant external assistance and resources, and where a multi-sectoral response is needed, with the engagement of a wide range of international humanitarian actors (IASC). This may include smaller-scale emergencies; in countries with limited capacities, the threshold will be lower than in countries with strong capacities. An emergency is a situation that threatens the lives and well-being of large numbers of a population and requires extraordinary action to ensure their survival, care and protection.”
2. Low and middle-income countries: We will define LMIC as countries defined by the World Bank as low-income, lower-middle income and upper-middle income. We will combine World Bank lists of LMIC countries from 2000 and 2020 to ensure we are inclusive of the vast majority of countries that have fit this definition in the specified time period.

**Nutrition outcomes:**

We broadly define nutrition outcomes as “outcomes of concern to nutrition actors.” Therefore, we include some outcomes that may result from a multiplicity of biological, psychosocial, behavioral and genetic factors, while recognising that the research that we include may not be able to adequately identify the strength of the connection between indirect exposure to IPV in-utero and the nutrition outcome. An example is low-birth weight, which may be multifactorial in origin, but for which risk may be exacerbated in the context of IPV, and thus would be relevant (and potentially actionable) for a nutrition actor .

1. **Inclusion and exclusion criteria:**

Studies will be eligible for inclusion in the review if the study:

- Utilizes any quantitative methodology; mixed methods studies will be included if quantitative findings are separately reported;
- Is published in peer-reviewed literature;
- Includes the following direct exposures for girls under the age of 18: child marriage, early marriage, forced marriage, preferential feeding, sexual violence, any form of intimate partner violence [IPV] (physical, sexual, psychological or controlling behaviours);
- AND/ OR includes the following indirect exposure for boys or girls under the age of 18: IPV against a maternal caregiver; including physical, sexual or psychological violence or controlling behaviors;
- Includes one or more of the following nutrition outcomes: Maternal iron deficiency anemia; Gestational diabetes; Maternal physical abdominal trauma; large for gestational age [LGA]; Small for gestational age [SGA]; Intrauterine growth restriction [IUGR]; Low birth weight; Delayed growth in infancy (affecting weight and or height); Below ten percentile weight; Below ten percentile height; Length for age; Risk for stunting and/or acute wasting; Any breastfeeding practices; Failure to thrive; Nutrient deficiencies; iron deficiency anemia in child; vitamin D deficiency/rickets; protein-energy malnutrition; *Risk for overweight/ obesity; Less healthy dietary practice;* underweight/ low body mass index; Low mid arm circumference;
- Includes girls for the direct pathways and child meaning boy or girl under the age of 18 for the indirect pathway; studies that include girls or girls and boys above the age of 18 as well must provide age-disaggregation such that outcomes for girls/ children below the age of 18 can be assessed;
- Is conducted in a low and middle-income context, or humanitarian context [to be determined through the course of the review].

Studies and data will be excluded from the review if they:

- Do not address the association between direct or indirect GBV and children’s nutrition outcomes (i.e. GBV and/ or nutrition are only included in analyses as control variables)
- Are grey literature, dissertations, presentations, book chapters
- Are a single case report
- Are published before 2000
- Are published in other languages that cannot be reviewed by the team

**Exposure(s)**

The REA has two distinct aims focused on exposure to different forms of GBV. The first concerns girls’ direct exposure to GBV and the second concerns children’s indirect exposure to IPV, through in-utero exposure (where a maternal caregiver experiences IPV while the child is in-utero) and household exposure (where the maternal caregiver experiences IPV, and the child is indirectly affected).

The types of **direct GBV** experienced by girls (aged under 18) that we will include are:

- Child marriage (including forced marriage of girls under 18 and early marriage)
- Preferential feeding of boys
- Sexual violence
- IPV

The types of **indirect GBV** experienced by children (girls and boys aged under 18) that we will include are all forms of IPV. There is considerable consensus concerning definitions of physical and sexual IPV: physical IPV is defined as “acts that can physically hurt the victim, including, but not limited to: being slapped or having something thrown at you that could hurt you; being pushed or shoved; being hit with a fist or something else that could hurt; being kicked, dragged or beaten up; being choked or burnt on purpose; and/or being threatened with or actually having a gun, knife or other weapon used on you,” and sexual IPV is defined as “ being physically forced to have sexual intercourse when you do not want to; having sexual intercourse out of fear for what your partner might do or through coercion; and/or being forced to do something sexual that you consider humiliating or degrading” [1]. There is less consensus concerning psychological violence (which may be referred to as verbal abuse or emotional violence) and controlling behaviours (which can include forms of economic violence). As such, we will include studies focusing on or including these types of IPV, and in the data extraction phase, indicate the definition that has been operationalised by the study authors.

**Nutrition-related exposures:**

In our scoping review, we identified considerable evidence concerning the associations between food insecurity and male perpetration of intimate partner violence and/ or female violence victimization. This pathway may be useful in understanding some of the interconnections between exposure to gender based violence and nutrition outcomes. However, these studies largely do not specifically assess children’s nutrition outcomes. Inclusion of nutrition or food insecurity related ***exposures*** is beyond the scope of the current review. However, when screening at the title/ abstract or full-text stage, we will tag manuscripts focusing on food insecurity as an exposure, compile a list of relevant articles, and draw on this in our findings and syntheses (for example, in proposing areas for future research). Food insecurity may occur as a result of GBV and may be an intermediary in the path from exposure to gender based violence and poor nutrition outcomes in children. When household food security is studied as an intermediate outcome, we will consider it for inclusion in our review.

**Main outcome(s):**

Outcomes have been selected based on important developmental milestones in children, by age, that are of concern to nutrition actors, and can impact nutrition programming.

**In utero:** We will separate the in utero outcomes into two categories that can impact overall outcomes for the child.

- **Maternal nutritional outcomes:** impact fetal growth and include maternal iron deficiency anemia, gestational diabetes. Here we will also include maternal infections resulting from GBV, resulting in SGA infants, as well as the maternal physical abdominal trauma which can result in SGA, or premature delivery.
- **Fetal nutrition related outcomes**: are large for gestational age, small for gestational age (SGA), intrauterine growth restriction (IUGR)

**Infancy (0-11.9 months):** low birth weight, delayed growth in infancy (affecting weight and or height) below ten percentile weight, <10% height, length for age, (weight for length), risk for stunting and/or acute wasting;  **Maternal:** suboptimal breastfeeding practices, with reduced/ shortened exclusive breast feeding and suboptimal timing for introduction of water or complementary feeding, initiation of breastfeeding within the first hour.

**0-2 years**: all of the factors affecting infancy, as well as failure to thrive, nutrient deficiencies iron deficiency anemia, vitamin D deficiency/rickets), protein-energy malnutrition, continued breastfeeding to 2 years; minimal dietary diversity, minimal meal adequacy, Bottle feeding 0–23 months; Continued breastfeeding 12–23 months; animal based protein; introduction of solid, semi-solid or soft foods; Sweet beverage or Unhealthy food consumption: age appropriate, nutrient dense foods.

**In pre-school children**: iron deficiency anemia (especially in setting of recurring infections including ascaris), risk for overweight/ obesity

**0-5 years:** all outcomes of interest to nutrition actors that impact children in infancy/ 0-2 years, as well as pre school children

**School age (5-11):** micronutrients underweight/ low body mass index, low mid arm circumference, nutrient deficiencies (vitamin D/rickets), protein-energy malnutrition, risk for overweight/ obesity; less healthy dietary practice/ habits (ie intake of sugary sweetened beverages, saturated fats, limited dietary diversity); risk for overweight/ obesity

**Adolescence (puberty to 18 years):** iron deficient anemia

We will also explore food security as an outcome. Since most food security measures used in population based studies capture food security at the household level, we will include the household food security score, as well as the specific scoring for children in the household if this is available for review. Household food security will serve as a proxy for child food security unless a specific youth /adolescent tool has been used. If an age specific tool has been used, we will use that measurement as an outcome instead.

Studies focusing on maternal nutrition outcomes (and not including any child-level outcomes) may be identified in the course of the review. We will generate a list of these studies, and at the point of data extraction and quality assessment, assess whether we can include these studies within the scope of the present REA. This does not represent a systematic search of GBV and maternal nutrition outcomes, however, may give an indication of the scope of the literature and potential future research questions focusing on maternal nutrition outcomes.

**Context**

One of the objectives of the REA is to build evidence for integrating GBV risk mitigation within nutrition programming in humanitarian settings. However, there may be very limited evidence for some of the aspects of the research objectives for the REA (based on our scoping review - primarily, for girls’ direct GBV exposure and nutrition outcomes). Moreover, some evidence may be useful for UNICEF’s work with vulnerable populations in non-emergency contexts. Given the limited literature, we will not include location/ context in the search strategy. While screening at the title/ abstract phase, we will tag and include for full text review studies conducted in LMIC. Depending on the breadth of included full texts, we will discuss as a research team and make a recommendation to UNICEF regarding how to proceed in terms of which contexts to include in the REA.

Based on our rapid scoping phase, the decision regarding context may differ according to the specific aim/ type of violence exposure - for example, there is more extensive literature on in-utero IPV exposure and nutrition outcomes than child marriage and nutrition outcomes.

We will draw on ***reviews*** of relevant questions that focus on or include studies in high-income contexts, including studies focusing on vulnerable populations in high-income contexts which may partially inform possible low and middle-income context pathways. Where necessary or particularly relevant to address an evidence-gap in our review, we will look at the original articles included in these reviews.

**Types of studies to be included:**

The REA will include quantitative studies. Any study that utilizes quantitative measurement methods - descriptive, correlational, quasi-experimental and experimental - will be included. The focus on quantitative research is in order to illustrate plausibility of different causal pathways identified in the review. We will explicitly tailor searches of databases to identify and include quantitative studies, however, where relevant qualitative studies are identified at the title/ abstract screening phase, we will tag these studies, generate a list of relevant qualitative studies, and where possible/ useful, draw on these studies in our final narrative synthesis to illustrate how particular pathways identified in the quantitative studies may operate.

Mixed methods studies will be included if quantitative findings are presented separately.

1. **Searches**

The following databases will be searched: Medline, Embase and Global Health. Search strategies developed for research aim 1 and 2 will be optimized for each database using both MeSH headings and keywords. The search results will be combined in Covidence and tagged by direct or indirect pathway. Duplicates will also be removed. In addition to the database search we will also extract primary studies from the reviews we identified in the scoping phase.

The search strategy will identify focus on the following domains presented in Table 1 and 2 (Appendix). (Note: MESH terms are optimized for Medline).

**Screening and selection**

All results from database searches will be imported into *Covidence* and duplicates will be removed. The screening and selection of included articles will occur in several stages:

*i. Title and abstract review:*

LV and SBC will both screen 10% of the title/ abstracts, with discussion of conflicts with a third member of the team. Following resolution of conflicts, and discussion, revision and refinement of the inclusion and exclusion criteria, LV and SBC will divide the remaining title/ abstract for screening.

Through the title/ abstract review process, articles will be tagged with the following tags:

-direct exposure

-indirect exposure

-humanitarian context

-LMIC context (non-humanitarian)

*ii. Full text review*

Once potential studies have been identified, full texts of selected abstracts will be obtained and screened by one reviewer. LV and SBC will screen all English language full text according to the inclusion/ exclusion criteria. Reasons for exclusion will be recorded, following an a priori, hierarchically determined exclusion list. A third author will resolve any disagreements. At this stage of the review, we will assess how many Spanish language articles fit our inclusion/ exclusion criteria, and team capacity to extract data and conduct quality assessment on these articles.

As screening progresses, the team will hold weekly meetings to resolve conflicts and discordance, discuss emerging patterns in the data and engage in a reflexive research process. At this point, depending on the number of included studies, the team will discuss scope of the review with UNICEF to ensure that a clear, defined and adequately circumscribed body of literature is utilized for the next steps. For example, at this stage, questions concerning where to include studies conducted in LMIC non-humanitarian contexts and where to narrow to humanitarian contexts will be discussed and resolved.

Specific tasks in this phase are subject to change depending on workload, i.e. a third team member may participate in title/ abstract screening or full text screening, to ensure smooth and rapid completion of this phase of the REA.

1. **Data extraction, quality assessment and data synthesis**

**Data extraction:**

We will develop a data extraction template in Covidence that addresses the key objectives of the REA. We will circulate a draft of the data extraction template to UNICEF colleagues for discussion, to ensure that key aspects of the evidence-base are being assessed and can be reported upon in the final analysis.

The data extraction process will focus on i) study design and methodology of included studies, ii) evidence of pathways between GBV and nutrition outcomes, iii) measures utilized for violence exposure and nutrition outcomes, and key moderators or mediators.

Examples of categories and related variables for data extraction include:

*Context:*

- Humanitarian context (yes/ no)
- Type of humanitarian context (i.e. precipitating factor(s) of humanitarian emergency - conflict, earthquake, typhoon, etc)
- Phase of humanitarian contexts (active crisis, protracted crisis, stabilization, return/ recovery)
- Living conditions (short-term emergency shelter; camp (refugee/ IDP), urban, informal settlement, etc)

*Study design:*

- Design
- Research question(s)
- Theoretical or conceptual framework - Y/N; If yes, describe
- Data collection methods
- Data analysis methods

*Sample:*

- Sample size
- Sampling procedure
- Age range - disaggregate by ages, etc.
- Inclusion/ exclusion

*Exposure (violence):*

- Type of violence
- Context of violence
- Perpetrators
- Measurement - name of scale, items
- Time frame

*Outcomes (nutrition):*

- Type of nutrition outcome
- Type of measure/ report (i.e. physical measurement, self-report)
- Direct or indirect association with exposure

**Risk of bias (quality) assessment**

We will use the 20-item [Appraisal tool for Cross-Sectional Studies](https://bmjopen.bmj.com/content/6/12/e011458) (AXIS tool) to assess the quality of cross-sectional studies. A summary of the AXIS tool is below:


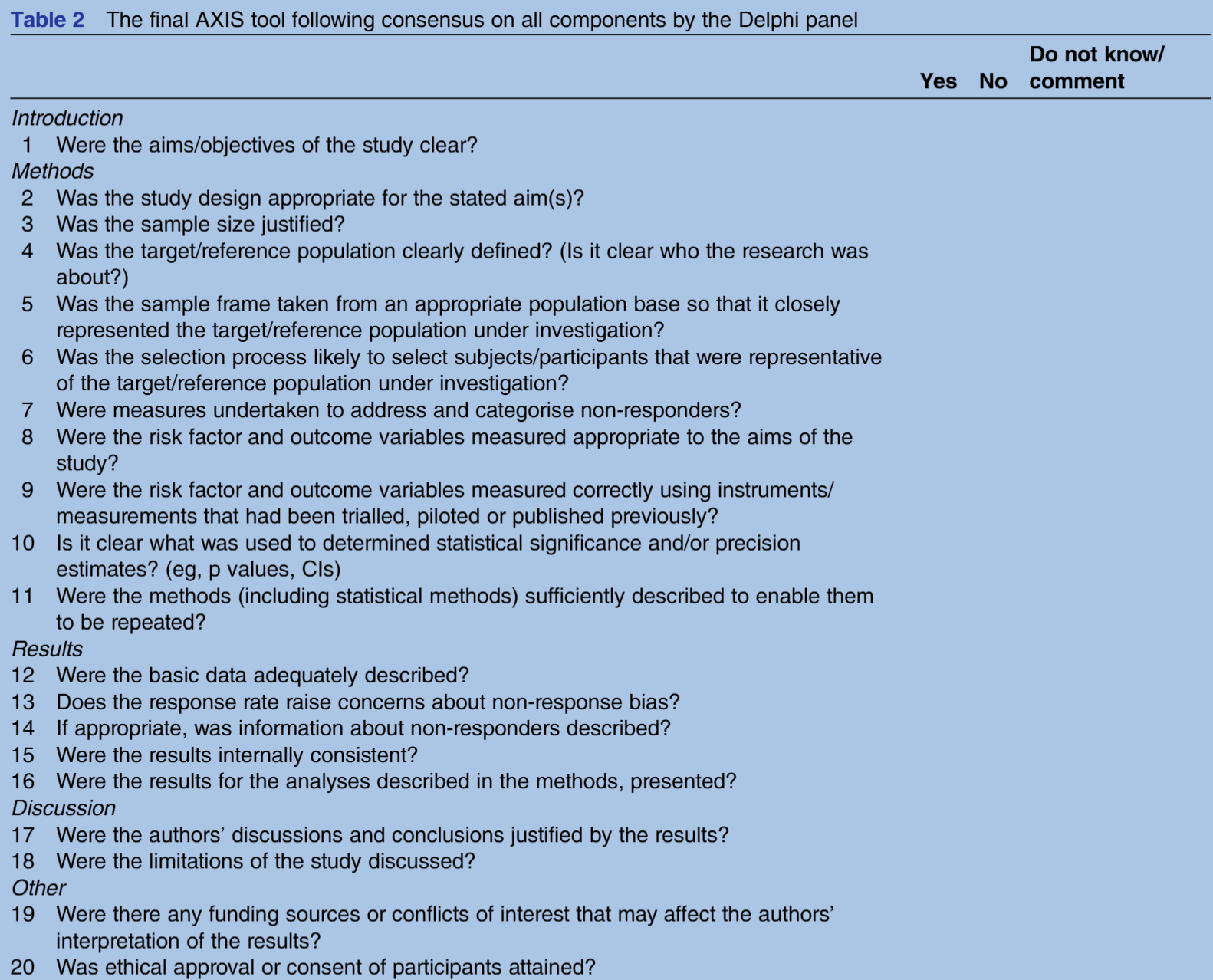


*From <https://bmjopen.bmj.com/content/6/12/e011458>

**Data synthesis**

Following data extraction and quality appraisal of all included articles, we will employ various stages of data analysis. The focus of our data analysis will be twofold:

i) To provide an in-depth description of the availability, quality and strength of evidence for the specific research aims, and if possible, disaggregated by other relevant factors (i.e. age group, types of nutrition outcomes, types of violence exposures); and

ii) To provide thematic synthesis of the evidence, according to plausible pathways between violence exposure (direct or indirect) and nutrition outcomes.

To provide an in-depth description of the evidence-base, we will conduct descriptive analysis of the data extracted from the included manuscripts, and generate tables focusing on i) study design, sample, research questions, ii) descriptive aspects of violence exposure (for example, measurement, type, perpetrators), iii) descriptive aspects of nutrition outcomes (for example, specific nutrition outcome, measurement method), and iv) plausible causal pathways/ associations described in the manuscripts. This stage of analysis will provide a snapshot of the included studies, and allow for insight into specific areas of stronger evidence and areas with evident gaps in data. This stage of the analysis is a form of narrative synthesis, which “involves collating study findings into a coherent textual narrative, with descriptions of differences in characteristics of the studies including context and validity, often using tables and graphs to display results” [2].

We will also conduct thematic synthesis of the included studies, categorised by which study aim they address, in order to identify the pathways (causal or correlational) in the evidence-base. Thematic synthesis is a form of synthesis often applied to qualitative systematic reviews [3]. In the case of our review, where we seek to identify patterns of associations and potential pathways between GBV and nutrition outcomes, this is an appropriate data synthesis methodology. In the context of this review, this approach will be adapted so that the texts being coded are not texts of study findings, as would be the case in qualitative systematic reviews, but are instead variables - exposures, outcomes, moderators and mediators - that can plausibly be linked to causal pathways and frameworks derived from our conceptual framework.

Steps of the thematic analysis include: i) developing the codebook through initial collaborative coding, ii) grouping, combining and refining codes, and iii) coding studies with the refined codebook [4]. In step 1, we will collaboratively develop a codebook of potential pathways, through team coding of a subset of included studies. Steps 2 and 3 will be implemented by sub-sets of the full research team, with review from other team members to ensure consistency and quality control. We will utilise the findings from the quality assessment to inform our interpretation of the certainty of specific findings.

We will follow some of the recommended steps in recently released guidelines regarding narrative synthesis without meta-analysis [5]. For example, we will present and explore:

- Grouping of studies for synthesis: depending on the evidence-base and findings in the REA so far, we will group studies by study design, population, age group or specific outcome in order to clearly present narrative syntheses of the included studies;
- Describe the synthesis methods: while we will not employ statistical methods to determine direction of effects or strength of correlations, we will clearly describe the coding procedure and combine the thematic analysis with our quality assessment procedure to provide an assessment of confidence in the pathways presented;
- Discussion of heterogeneity: we will explore heterogeneity of effect sizes, directions of correlations and associations, and strength of associations; and
- Certainty of evidence: we will report our findings from the quality assessment to present certainty of evidence on specific pathways.

We do not plan to do any meta-analysis or other statistical analyses on the outcomes of the included studies. These approaches are more appropriate for systematic reviews focused on intervention (i.e. meta-analysis of intervention effects).
